# Market survey on the traditional medicine of the Lijiang area in Yunnan Province, China

**DOI:** 10.1186/s13002-022-00532-w

**Published:** 2022-05-23

**Authors:** Mingshuo Zhang, Haitao Li, Junqi Wang, Maohong Tang, Xiaobo Zhang, Shaohua Yang, Jianqin Liu, Ying Li, Xiulan Huang, Zhiyong Li, Luqi Huang

**Affiliations:** 1 School of Pharmacy, Minzu University of China, Beijing, 100081 China; 2 Lijiang Medical Association of Minorities, Lijiang, 674100 China; 3 Yunnan Key Laboratory of Southern Medicinal Resources, Yunnan Branch, Institute of Medicinal Plant Development, Chinese Academy of Medical Sciences and Peking Union Medical College, Jinghong, 666100 China; 4 Chinese Medicine Resource Center of China Academy of Chinese Medical Sciences, Beijing, 100700 China; 5 Institute of Alpine Economic Botany, Yunnan Academy of Agricultural Sciences, Lijiang, 674100 China; 6 Lijiang Dongba Culture Research Institute, Lijiang, 674100 China; 7 Institute of Chinese Materia Medica, China Academy of Chinese Medical Sciences, Beijing, 100700 China; 8 China Academy of Chinese Medical Sciences, Beijing, 100700 China

**Keywords:** Ethnobotany, Lijiang area, Medicinal plants, Traditional knowledge, Medicinal market, Naxi, *Dongba Sutra, Yulong Ben Cao*

## Abstract

**Background:**

Traditional markets are important trading places for medicinal plants, and researchers performing market surveys often engage in ethnobotanical research to record the herbal plants used locally and any related traditional knowledge. However, information on market-traded medicinal plants from traditional markets in the Lijiang area of Yunnan is not well documented. This research is an ethnobotanical survey focusing on medicinal plants traded in the traditional markets of the Lijiang area and contributes to the understanding of medicinal plants and related information used by the Naxi people.

**Methods:**

Ethnobotanical surveys were performed for two years (2019–2020). Three traditional markets in the Lijiang area were investigated. The methods we used included literature research, participatory surveys and group discussions. The collected voucher specimens were identified using the botanical taxonomy method and were deposited in the herbarium. The data were analysed through the informant consensus factor and use frequency (UF). These medicinal plants were compared with the Information System of Chinese Rare and Endangered Plants from the Chinese Academy of Sciences. Those results were in turn compared with the Dongba Sutras and *Yulong Ben Cao*.

**Results:**

A total of 277 species from 97 families were recorded, with Asteraceae providing the maximum numbers of medicinal plants. Among them, 248 species (89%) were wild plants and 266 species (92.39%) were from the local area. Root (40.43%) was the most common medicinal part. A total of 267 species (96.04%) had a UF value above 0.5. Eighty-three investigated human ailments were grouped into 16 categories. Diseases of the digestive system (166 mentions) were most frequently mentioned in this study. There were 19 species of nationally protected plants in China, including 2 species of first-level nationally protected plants and 17 species of second-level nationally protected plants. A total of 31 species of these medicinal plants can be found in the *Dongba Sutra* or *Yulong Ben Cao*.

**Conclusion:**

We surveyed the herbal medicine in the markets covering the Lijiang area, analysing and revealing the resource composition and current market situations. The medicinal plants used by the Naxi people are diverse and are used to treat a wide spectrum of body disorders. There are many wild medicinal plants, and to ensure sustainable development, their natural protection should be strengthened. Knowledge of the medicinal plants recorded in Naxi medical classics has ethnobotanical value and should be further developed.

## Background

Herbal medicines have played a distinctive role from the primitive period until today in health care systems [1–3]. Approximately 80% of the global population currently uses traditional herbal medicines [4, 5]. These herbal medicines have been used for more than 5000 years in China, and their development is also highly valued there [3]. From 2011 to 2020, China implemented its Fourth National Survey of Chinese Materia Medica Resources to improve the management of these resources [6]. To tap the modern value of ethnic medicine fully and attain a sustainable use of resources, a survey of traditional knowledge related to ethnic medicine was performed [7]. The Lijiang area started the fourth survey of Chinese medicinal resources in 2011. As of June 2021, the 2060 specimens of Chinese medicinal materials had been collected and identified; among them, 85 species of herbs recorded in the Dongba Sutra had been identified [8].

In areas with abundant ethnobotanical knowledge, market surveys are an important research method for ethnobotanical research on medicinal plants [9]. Many studies of traditional herbal markets have been conducted; for example, a study of medicinal plants sold in traditional markets in southern Ecuador found 160 medicinal plants in 57 families and identified 11 culturally significant medicinal plants according to their fidelity level (FL) [10]. An ethnobotanical survey of medicinal plant species marketed in Mashhad city, north-eastern Iran was conducted to document traditional medicinal knowledge and the application of medicinal plants [11]. Market research in Yunnan Province, China, found that herbs collected at the Dragon Boat Festivals in China are considered to be of higher quality than those collected at other times [9, 12]. In China, traditional markets are considered important places for the trading of medicinal plants harvested by rural villagers, and they also play a social role in exchanging the traditional use of herbal medicine among different cultural and social groups in local areas [13].

The Naxi people are primarily distributed in Yunnan and Sichuan Provinces and the Tibet Autonomous Region. Among the total population, more than two-thirds of the Naxi live in Lijiang, Yunnan Province [14]. The Lijiang area is located in the Hengduan Mountains, and it has a fertile soil, a suitable climate, a forest coverage rate of 70% and rich resources in terms of Chinese medicinal materials; it is known as the "hometown of medicinal materials". More than 500 species of medicinal herbs grow on Yulong Snow Mountain, which is known as the "treasure house of plants" [15]. The Naxi people are a nation with a long history in China whose ancestors created the splendid Dongba culture. The Dongba Sutra involved many traditional disciplines, such as history, philosophy, religion, music, dance, etc., and it also recorded a great deal of medical knowledge, including *Genesis* and *Chongren Pandi to Find Medicine*. Naxi Dongba medicine is a traditional medicine that has been used by the Naxi people for generations to prevent and treat diseases. The Naxi ancestors accumulated a wealth of knowledge and medical literature [16].

Thus far, there has been a lack of ethnobotanical research on the traditional medicinal plant knowledge of the Naxi people in the Lijiang area. Therefore, based on the theories and methods of ethnobotany, we investigated the medicinal plants of the Naxi people in the Lijiang area to address three objectives: (1) to document traditional knowledge of ethnic medicinal plants in the Lijiang area, (2) to identify potential conservation threats and (3) to record the medicinal plants sold on the market from medical classics.

## Materials and methods

### Study area

**Fig. 1:**
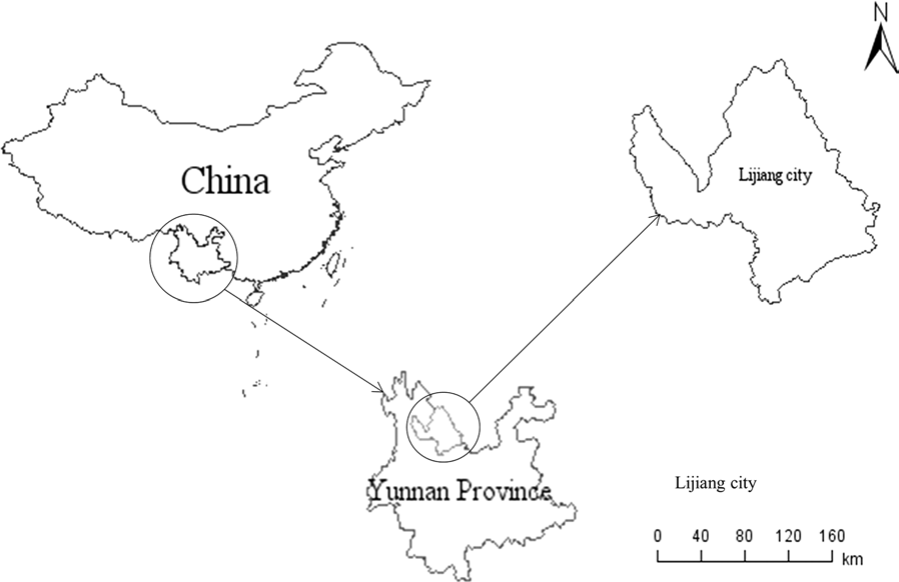
Location of the Lijiang area, Yunnan Province, China

**Fig. 2:**
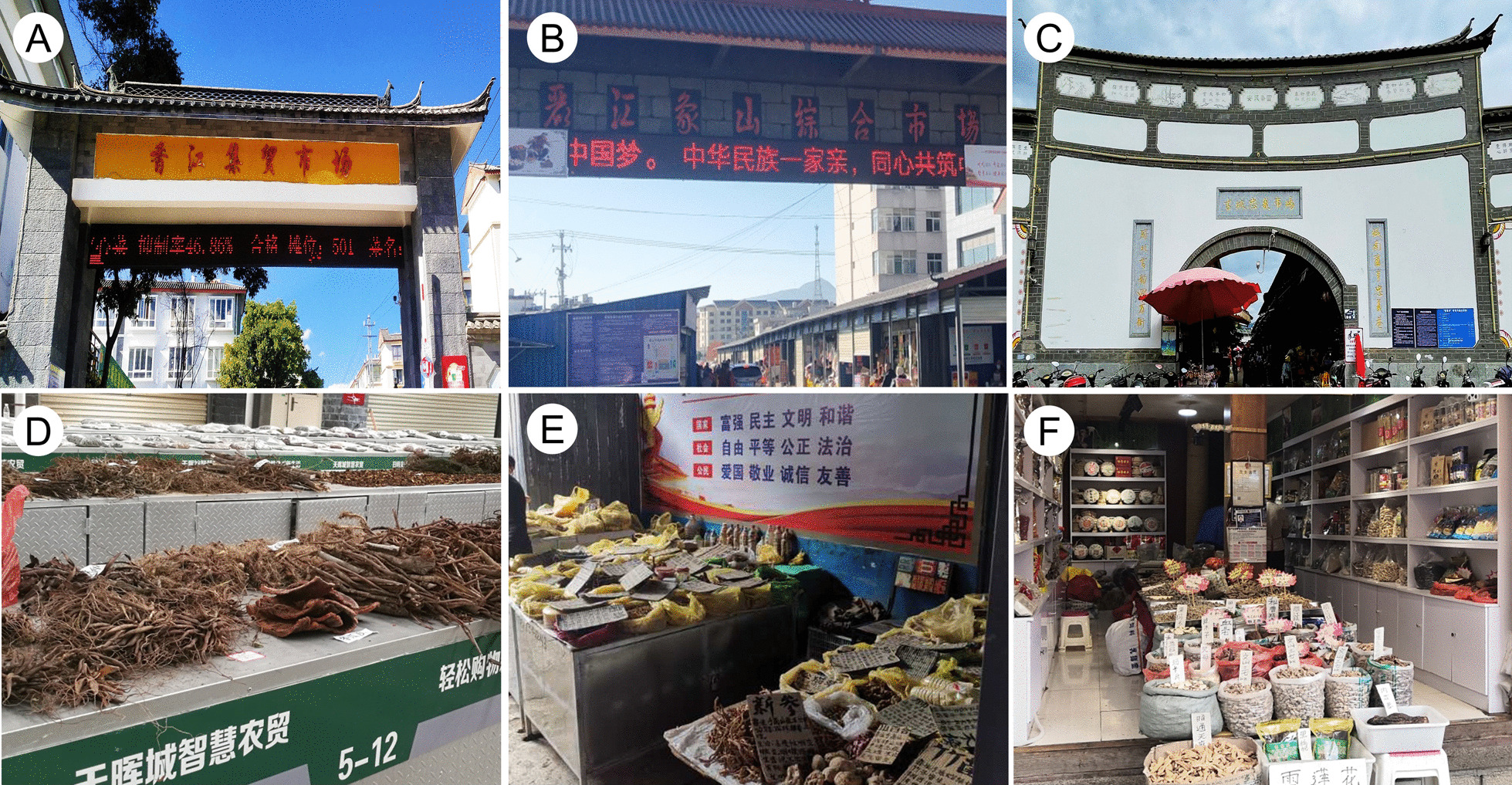
The herb trading markets in the Lijiang area (**A**, **D** Xiangjiang Market; **B**, **E** Xiangshan Market; **C**, **F** Zhongyi Market)

The Lijiang area is located in north-western Yunnan Province and the city centre is located at E100°25′ and N26°86′, with a total area of 20,600 km^2^ (Fig. 1). Apart from the Han nationality, there are 22 ethnic minorities living in Lijiang, among which two-thirds of the Naxi people live in Lijiang. The Lijiang area is near to the Hengduan Mountains, with alternating mountains, river valleys and tableland. The soil is fertile, there are many hours of sunlight and there is abundant rainfall [17]. Chinese medicinal materials are very rich in resources. Here we investigated three markets in Lijiang, namely Zhongyi Market, Xiangshan Market and Xiangjiang Market (Fig. 2).

### Data collection

From August 2019 to December 2020, we completed an investigation of the three medicinal material trading markets in the Lijiang area. The methods that we adopted included literature research, participatory investigation and group discussions. We recorded and photographed the medicinal plants sold in these markets. The collected samples were identified by the taxonomists Haitao Li and Shaohua Yang, according to the Flora of China [18] and Flora Yunnanica [19], who undertook census tasks for Yunnan and Lijiang area. The samples were preserved at the Institute of Alpine Economics and Botany, Yunnan Academy of Agricultural Sciences, which is the main storage targets for the general survey of Chinese medicine resources in Lijiang (Codes of Voucher Specimens: NXYC001-NXYC277).

Eighteen folk doctors in Lijiang were interviewed to investigate the application of the medicinal materials. The uses of medicines were organized according to the International Classification of Primary Care (ICPC-2) [20]. According to the results of the fourth national survey of traditional Chinese medicine resources, whether the medicinal plants sold in the markets came from local or other places, wild or cultivated should be indicated. The Information System of Chinese Rare and Endangered Plants (ISCREP) [21] was used to check whether these medicinal plants belonged to the protected plants.

The Dongba Sutras is a special scripture and is different from the Buddhist Sutras or other classics. The contents of the Dongba Sutras cover history, philosophy, society, religion, language and script, music, art, dance and many other traditional subjects related to Dongba culture. It is praised in academic circles as “the encyclopedia of ancient Naxi people” [22]. Naxi medical culture is an important part of Dongba culture. The Dongba Sutras contain information about the unique medical culture of the Naxi people, and they are the most important documents for studying Naxi medicine. The *Yulong Ben Cao* was first created during the Ming Dynasty and was written during the Qing Dynasty. It was written in Chinese by Jieshan He, a Naxi person. It was a local herbal work written under the guidance of Traditional Chinese Medicine (TCM) theory and his personal experience in applying medicines from the Naxi people. It is the product of the combination of Naxi culture and Han culture [23]. Therefore, the medicines from the markets were consulted using these works.

### Data analysis

The data collected in this study were analysed and graphed by using Microsoft Office Excel (2010) and RStudio software (4.1.1), including the informant consensus factor (ICF) and use frequency (UF). A quantitative data analysis was conducted using the ICF method and the number of citations. The ICF was calculated as the ICF = (Nur − Nt)/(Nur − 1), where Nur is the sum of plant species used by all the respondents to treat a particular disease and Nt is the number of identical plant species used by all the respondents to treat a particular disease [24]. The use frequency of medicine sold on the market was estimated with the utilization frequency [25]. The UF is calculated as UF = Nm/Ni, where Nm is the number of use reports of medicinal materials mentioned by informants, and Ni is the total number of informants. High UF values indicate that the herb is used more frequently in the region.

## Results and discussion

### Floristic diversity and UF

**Fig. 3:**
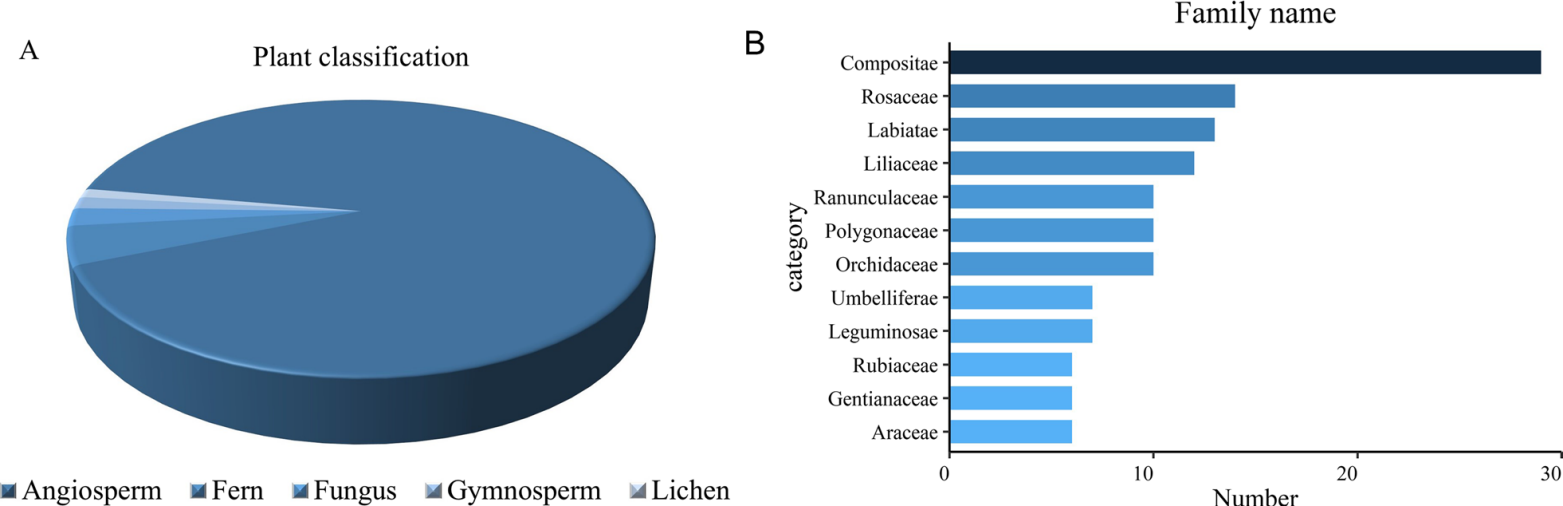
**A** Taxonomic composition and percentage of 277 plant medicines. **B** Dominant medicinal plant families recorded in 3 traditional markets

In the market, we encountered 277 species of medicinal plants whose base sources have been identified (Table 1). Among the 277 medicinal individuals, the original samples include fungi, lichens, bryophytes, ferns, gymnosperms and angiosperms, of which angiosperms were the most diverse, accounting for 91% (Fig. 3A). The plants belonged to 97 taxonomic families according to the Flora of China and the Flora Yunnanica. The dominant plant family was Asteraceae, with 31 species representing 11.12% of the total species, followed by 14 species of Rosaceae and Lamiaceae (5.05%) and 10 species of Orchidaceae and Polygonaceae (3.61%) (Fig. 3B). Other studies on traditional markets of the Naxi people of the Lijiang area also recorded Asteraceae as the family with the highest number of medicinal plant species, and there were many varieties [26, 27]. This result indicated that the Naxi medicinal plants in Lijiang involved a wide range of families, which was consistent with the rich plant resources and biodiversity in Lijiang, indicating that the Naxi people in Lijiang had a degree of systematic and comprehensive understanding and use of these medicinal plants. Combined with the fourth national survey of traditional Chinese medicine resources, among the 277 species of medicinal plants, 133 species (48.01%) were found to be distributed in the city, 54 species (19.49%) were distributed along the Jinsha River, and 14 species (5.05%) were distributed on Yulong Snow Mountain, including *Rhodiola fastigiata*, *Pyrola forrestiana*, *Aconitum brachypodum*, etc.

**Table 1:** The 277 species of medicinal plants in this study and their relevant information

Voucher Specimens	Scientific name	Local name	Family name	Medicinal parts	W/C	L/E	Diseases treated (ICPC-2)	UF
NXYC242	*Cordyceps sinensis* (Berk.) Sacc	Chong cao	Clavicipitaceae	Others	W	L	Anemia B82, lmpotence NOS Y07, Tuberculosis A70, Haematemesis D14	0.94
NXYC145	*Cryptoporus volvatus* (Peck) Huhhara	Se hei	Poliporaceae	Fruitbody	W	L	Asthma R96, Bronchitis R78, Hemorrhoids K96	0.72
NXYC119	*Engleromyces goetzii* P.Henn	Me mu	Hypocreaceae	Fruitbody	W	L	Malignant Neoplasms Stomach D74, Upper Respiratory Infection R74, Burns/Scald S14, Laryngitis R77, Tonsillitis R76, Pain General/Multiple Sties A01, Skin Disease Other S99, Neoplasm A21, Chronic Enteritis/Ulcerative Colitis D94, Parotitis R99	0.89
NXYC027	*Ganoderma applanatum* (Pers.)	Ping gai ling zhi	Ganodermataceae	Fruitbody	W	L	Foreign Body Nose/Larynx/Bronchus R87, Laryngitis R77	0.72
NXYC192	*Polyporus umbellatus* (Pers.) Fries	Zhu ling	Poliporaceae	Sclerotium	W	L	Malaria A73, Pyelonephritis U70	0.61
NXYC048	*Poria cocos* (Schw.)Wolf	Tuo ken lv	Poliporaceae	Sclerotium	W	L	Dyspepsia D07, Sleep Disturbance P06, Influenza R80	0.94
NXYC115	*Thamnolia vermicularis* Schaer	Guo lei	Compositae	Whole plant	W	L	Tuberculosis A70, Laryngitis R77, Epilepsy N88, Neurasthenia P78	0.89
NXYC053	*Usnea longissima* Ach	Ze fu	Usneaceae	Whole plant	W	L	Tuberculosis A70, Palpitation K04, Menstruation Irregular/Frequent X07, Dysuria U01, Sleep Disturbance P06, Rheumatoid Arthritis L88, Influenza R80, Bronchitis R78, Diarrhea D11, Abdominal Pain Epigastric D02, Hemiplegia N91, Breast Symptom Female other X21, Skin Disease Other S99	0.83
NXYC246	*Rhodobryum roseum* (Hedw.) Limpr	Nu mei lei ju ru	Bryaceae	Whole plant	W	L	Neurasthenia P78	0.78
NXYC159	*Lycopodium japonicum* Thunb. ex Murray	Yan tou zhi	Lycopodiaceae	Whole plant	W	L	Hepatitis D71, Cholelithiasis D98, Cataract F92, Neurasthenia P78, Conjunctivitis F70, Rheumatoid Arthritis L88, Pain General/Multiple Sties A01	0.56
NXYC034	*Selaginella pulvinata* (Hook. et Grev.) Maxim	Ci lv lv ru da biao	Selaginellaceae	Whole plant	W	L	Bleeding/Haemorrhage Nos A10, Menstruation Irregular/Frequent X07, Pain General/Multiple Sties A01, Liver Disease NOS D97, Rheumatoid Arthritis L88, Asthma R96	0.72
NXYC004	*Equisetum hyemale* L	Miao kong suo suo men	Equisetaceae	Whole plant	W	L	Jaundice D13, Hepatitis D71, Cataract F92, Bronchitis R78, Urinary Infection U71, Urinary Calculi U95, Hemorrhoids K96, Bleeding/Haemorrhage Nos A10	0.89
NXYC055	*Botrychium lanuginosum* Wall	Bai jie	Ophioglossaceae	Root	W	L	Influenza R80, Post-partum Symptom/Complaint other W18, Tuberculosis A70, Cataract F92, Sinusitis R75, Whooping Cough R71, Bronchitis R78, Skin Disease Other S99, Trauma A80	0.78
NXYC220	*Botrychium daucifolium* Wall	Mao chong lou	Ophioglossaceae	Whole plant	W	L	Cough R05, Skin Disease Other S99, Neoplasm A21	0.61
NXYC084	*Cibotium barometz* (L.) J.Smith	Gou ji	Cibotiaceae	Root	W	E	Rheumatoid Arthritis L88, Skin Disease Other S99, Pain General/Multiple Sties A01	0.83
NXYC134	*Adiantum philippense* L	Ge di li	Pteridaceae	Whole plant	W	L	Hepatitis D71, Urinary Infection U71, Pyelonephritis U70, Urinary Calculi U95, Burns/Scald S14	0.67
NXYC127	*Haplopteris flexuosa* (Fée) E. H. Crane	Hua shi cao	Pteridaceae	Leaf	W	L	Rheumatoid Arthritis L88, Cataract F92, Pain General/Multiple Sties A01, Bleeding/Haemorrhage Nos A10	0.78
NXYC210	*Davallia trichomanoides* Blume	Run hua jin cun	Davalliaceae	Rhizome	W	E	Hepatitis D71, Pyelonephritis U70, Pain General/Multiple Sties A01	0.5
NXYC093	*Drynaria delavayi* Christ	Lu ba di li	Polypodiaceae	Stem	W	L	Deafness H86, Pain General/Multiple Sties A01, Teeth/Gum Disease D82, Appendicitis D88	0.83
NXYC128	*Lepisorus bicolor* (Takeda) Ching	Liang se wa wei	Polypodiaceae	Leaf	W	L	Cough R05, Pyelonephritis U70, Burns/Scald S14	0.39
NXYC097	*Pyrrosia davidii* (Baker) Ching	Piao tan fu	Polypodiaceae	Whole plant	W	L	Urinary Calculi U95, Bronchitis R78	0.83
NXYC223	*Pinus yunnanensis* Franch	Ge bo ha	Pinaceae	Pollen	W	L	Skin Disease Other S99, Influenza R80, Worms/other Parasites D96, Skin Disease Other S99	0.61
NXYC047	*Platycladus orientalis* (L.) Franco	Bian bai	Cupressaceae	Fruit	W, C	L	Sleep Disturbance P06, Heart Pain K01, Constipation D12	0.94
NXYC051	*Ephedra likiangensis* Florin	Mie ku sa	Ephedraceae	Stem	W	L	Influenza R80, Pneumonia R81, Pyelonephritis U70, Bronchitis R78	0.83
NXYC135	*Kadsura coccinea* (Lem.) A. C. Smith	Leng fan tuan	Schisandraceae	Stem	W	E	Post-partum Symptom/Complaint other W18, Rheumatoid Arthritis L88, Menstruation Irregular/Frequent X07, Pain General/Multiple Sties A01, Abdominal Pain Epigastric D02, Chronic Enteritis/Ulcerative Colitis D94	0.61
NXYC156	*Schisandra neglecta* A.C.Smith	Xiao xue teng	Schisandraceae	Root	W	L	Pain General/Multiple Sties A01, Influenza R80	0.72
NXYC049	*Schisandra rubriflora* (Franch.) Rehd.et Wils	Guo ji lv	Schisandraceae	Stem	W	L	Rheumatoid Arthritis L88, Bronchitis R78, Hepatitis D71, Neurasthenia P78	0.83
NXYC179	*Houttuynia cordata* Thunb	A ruo ken	Saururaceae	Whole plant	W, C	L	Skin Disease Other S99, Tuberculosis A70	0.89
NXYC256	*Peperomia dindygulensis* Miq	Chi e a men	Piperaceae	Flower	W	L	Pyelonephritis U70, Skin Disease Other S99, Pain General/Multiple Sties A01, Cough R05, Rash Localized S07, Neoplasm A21	0.56
NXYC082	*Asarum himalaicum* Hook.f.et Thoms.ex Klotzxch		Aristolochiaceae	Whole plant	W	L	Influenza R80	0.72
NXYC107	*Magnolia delavayi* Franch	Ye he	Magnoliaceae	Flower, Bark	W	L	Bronchitis R78, Sinusitis R75	0.94
NXYC203	*Cinnamomum cassia* Presl	Se bi	Lauraceae	Bark	C	E	Menstruation Irregular/Frequent X07, lmpotence NOS Y07, Palpitation K04, Abdominal Pain Epigastric D02, Abdominal Hernia other D91, Heart Pain K01	0.89
NXYC088	*Cinnamomum tamala* (Buch.-Ham.) Nees et Eberm	Se bi	Lauraceae	Bark	W	L	Diarrhea D11, Menstruation Irregular/Frequent X07, Rheumatoid Arthritis L88, Pain General/Multiple Sties A01, Skin Disease Other S99, Bleeding/Haemorrhage Nos A10	1
NXYC002	*Acorus calamus* L	Chong beng	Acoraceae	Root	W	L	Epilepsy N88, Deafness H86, Rheumatoid Arthritis L88, Dementia P70	0.94
NXYC036	*Acorus gramineus* Soland	Lu chong ben	Acoraceae	Root	W	L	Epilepsy N88, Dementia P70, Deafness H86, Rheumatoid Arthritis L88, Asthma R96, Pain General/Multiple Sties A01, Skin Disease Other S99, Peptic Ulcer D86, Diarrhea D11	0.94
NXYC255	*Acorus tatarinowii*	Lu chong ben	Acoraceae	Rhizome	W	L	Epilepsy N88, Sleep Disturbance P06, Abdominal Pain Epigastric D02, Influenza R80, Skin Disease Other S99, Pain General/Multiple Sties A01, Stroke K90	0.67
NXYC064	*Pinellia ternata* (Thunb.) Breit	Ri ha	Araceae	Root, Fruit	W	L	Neoplasm A21, Breast Symptom Female other X21, Influenza R80, Skin Disease Other S99, Stroke K90	0.89
NXYC199	*Pinellia ternate* (Thunb.) Breitenb	Ri han	Araceae	Tuber	W	L	Carbuncle S10, Epilepsy N88, Neoplasm A21, Stroke K90, Trauma A80, Tetanus N72	0.94
NXYC264	*Typhonium divaricatum* (L.) Decaisne	Gou ban xia	Araceae	Tuber	W	L	Boil Carbuncle S10, Pain General/Multiple Sties A01, Neoplasm A21, Skin Disease Other S99, Trauma A80	0.78
NXYC219	*Paris polyphylla* Smith var. yunnanensis (Franch.) Hand.-Mzt	Yu ma pu	Melanthiaceae	Rhizome	W, C	L	Tonsillitis R76, Abdominal Pain Epigastric D02, Tuberculosis A70, Breast Symptom Female other X21, Trauma A80, Skin Disease Other S99	0.78
NXYC110	*Paris polyphylla* var. yunnanensis (Franch.) Hand.-Mazz	Sao xiu	Melanthiaceae	Root	W, C	L	Laryngitis R77, Stroke K90, Migraine N89, Trauma A80, Pain General/Multiple Sties A01, Skin Disease Other S99	0.78
NXYC267	*Veratrum nigrum* L	Ji ceng dao	Melanthiaceae	Root	W	L	Malaria A73, Epilepsy N88,Boil Carbuncle S10, Stroke K90	0.61
NXYC266	*Iphigenia indica* (L.) Kunth	Yi pi jian	Colchicaceae	Bulb	W	L	Foreign Body Nose/Larynx/Bronchus R87, Asthma R96, Bronchitis R78, Breast Lump/Mass Female X19	0.72
NXYC095	*Smilax glabra* Roxb	Chong ma	Smilacaceae	Root	W	L	Syphilis Y70, Skin Disease Other S99, Hemorrhoids K96, Prostatitis Y73, Rheumatoid Arthritis L88, Menstruation Irregular/Frequent X07, Neoplasm A21	0.89
NXYC120	*Smilax menispermoidea* A. DC	Lao ci	Smilacaceae	Stem	W	L	heumatoid Arthritis L88, Bronchitis R78, Skin Disease Other S99, Neoplasm A21, Syphilis Y70	0.67
NXYC218	*Fritillaria thunbergii* Miq	Bei mu	Liliaceae	Bulb	C	E	Upper Respiratory Infection R74, Goitre T81, Peptic Ulcer D86, Tuberculosis A70	0.61
NXYC224	*Bletilla striata* (Thunb.) Rchb. f	Gong ben ya de	Orchidaceae	Root	W	L	Tuberculosis A70, Whooping Cough R71, Bleeding/Haemorrhage Nos A10, Bronchitis R78, Skin Disease Other S99, Burns/Scald S14	0.94
NXYC043	*Bulbophyllum odoratissimum* (Smith) Lindl	Lv ji piao	Orchidaceae	Whole plant	W	L	Tuberculosis A70, Laryngitis R77, Pain General/Multiple Sties A01, Rheumatoid Arthritis L88	0.5
NXYC140	*Calanthe tricarinata* Lindl.ex Wall	A dong ming	Orchidaceae	Root, Fruit	W	L	Rheumatoid Arthritis L88, Abdominal Pain Epigastric D02, Pain General/Multiple Sties A01	0.39
NXYC175	*Dendrobium officinale* Kimura et Migo	Ai shi ban mi ba	Orchidaceae	Stem	W	L	Diabetes Mellitus T90	0.78
NXYC177	*Gastrodia elata* Blume	Lei ke	Orchidaceae	Tuber	W, C	L	Hypertension K25, Epilepsy N88, Stroke K90	0.89
NXYC162	*Goodyera repens* (L.) R. Br	Xiao ban ye lan	Orchidaceae	Whole plant	W	L	Skin Disease Other S99, Tuberculosis A70, Influenza R80, Pain General/Multiple Sties A01, Trauma A80	0.61
NXYC236	*Gymnadenia conopsea* (L.) R. Br	A yu la ba	Orchidaceae	Tuber	W	L	Hepatitis D71, Tuberculosis A70, Cough R05, Post-partum Symptom/Complaint other W18, Abortion Spontaneous W82, lmpotence NOS Y07, Menstruation Irregular/Frequent X07, Pain General/Multiple Sties A01, Diarrhea D11	0.67
NXYC237	*Gymnadenia orchidis* Lindl	Xi nan shou shen	Orchidaceae	Tuber	W	L	lmpotence NOS Y07, Menstruation Irregular/Frequent X07, Post-partum Symptom/Complaint other W18, Tuberculosis A70	0.61
NXYC225	*Pleione bulbocodioides* (Franch.) Rolfe	Gong ben ya ji	Orchidaceae	Bulb	W	L	Skin Disease Other S99, Neoplasm A21, Trauma A80	0.89
NXYC247	*Spiranthes sinensis* (Pers.) Ames	Lu bu gei	Orchidaceae	Flower	W	L	Herpes Zoster S70, Tuberculosis A70, Diabetes Mellitus T90, Haematemesis D14, Hypertension K25, Tonsillitis R76, Skin Disease Other S99, Vaginal Discharge X14	0.56
NXYC102	*Belamcanda chinensis* (L.) Redoute	Beng de piao ba	Iridaceae	Root	W	L	Cough R05, Urinary Calculi U95, Menstruation Irregular/Frequent X07, Hepatitis D71	0.83
NXYC241	*Crocus sativus* L	Zang hong hua	Iridaceae	Flower	W	L	headache N01, Bleeding/Haemorrhage Nos A10, Menstruation Irregular/Frequent X07, Pain General/Multiple Sties A01, Liver Disease NOS D97	0.83
NXYC033	*Asparagus filicinus* Ham. ex D. Don	Sui ben mei jiu	Asparagaceae	Root	W	L	Tuberculosis A70, Whooping Cough R71, Asthma R96, Laryngitis R77, Rheumatoid Arthritis L88, Boil Carbuncle S10	0.94
NXYC232	*Ophiopogon japonicus* (Thunb.) Ker Gawl	Mai dong	Asparagaceae	Tuber	W	L	Cough R05, Tuberculosis A70	0.61
NXYC235	*Polygonatum cirrhifolium* (Wall.) Royle	Mei zi na wang bo	Asparagaceae	Rhizome	W	L	Cough R05, Diabetes Mellitus T90, lmpotence NOS Y07, Menstruation Irregular/Frequent X07, Laryngitis R77, Dyspepsia D07, Neoplasm A21, Vaginal Discharge X14	0.78
NXYC015	*Polygonatum kingianum* Coll. et Hemsl	Mei zi na wang bo	Asparagaceae	Root	W	L	Hepatitis D71, Tuberculosis A70, Cough R05	0.83
NXYC117	*Reineckea carnea* (Andrews) Kunth	Jiu jie ling	Asparagaceae	Root	W	L	Cough R05, Pyelonephritis U70, Tuberculosis A70, Bronchitis R78, Whooping Cough R71, Trauma A80, Breast Symptom Female other X21, Rheumatoid Arthritis L88, Menstruation Irregular/Frequent X07, Cystitis U71, Chronic Enteritis/Ulcerative Colitis D94, Pain General/Multiple Sties A01	0.72
NXYC151	*Trachycarpus fortunei* (Hook.) H. Wendl	Ji ce lv	Arecaceae	Fruit	W	L	Bleeding/Haemorrhage Nos A10, Rheumatoid Arthritis L88, Pain General/Multiple Sties A01, Boil Carbuncle S10, Vaginal Discharge X14	0.56
NXYC250	*Cyanotis arachnoidea* C. B. Clarke	Lu shui cao	Commelinaceae	Flower	W	L	Dysuria U01, Skin Disease Other S99, Rheumatoid Arthritis L88	0.44
NXYC007	*Alpinia officinarum* Hance	Gu shu	Zingiberaceae	Root	W	L	Vomiting D10, Dyspepsia D07, Malaria A73, Chronic Enteritis/Ulcerative Colitis D94	0.89
NXYC041	*Alpinia zerumbet* (Pers.) Burtt. et Smith		Zingiberaceae	Fruit	W	E	Chronic Enteritis/Ulcerative Colitis D94	0.78
NXYC100	*Hedychium spicatum* Buch.-Ham. ex Smith	Gu lao	Zingiberaceae	Rhizome	W	L	Cough R05, Dyspepsia D07, Pain General/Multiple Sties A01, Question of Pregnancy W01, Abdominal Pain Epigastric D02, Rheumatoid Arthritis L88	0.94
NXYC013	*Zingiber officinale* Rosc	Gu bu	Zingiberaceae	Root	C	L	Influenza R80, Vomiting D10, Rheumatoid Arthritis L88, Menstruation Irregular/Frequent X07, Cough R05, Diarrhea D11, Pain General/Multiple Sties A01, Poisoning by Medical Agent A84	1
NXYC094	*Juncus effusus* L	Ji beng ken	Juncaceae	Whole plant	W	L	Influenza R80, Urticaria S98, Syphilis Y70, Jaundice D13, Pyelonephritis U70, Prostatitis Y73, Dysuria U01, Burns/Scald S14, Pneumonia R81, Malaria A73	0.94
NXYC008	*Cyperus rotundus* L	Si gua ri	Cyperaceae	Root	W	L	Heart Pain K01, Menstrual Pain X02, Dyspepsia D07, Cough R05	0.94
NXYC124	*Cymbopogon distans* (Nees ex Steud.) Wats	Xiang mao cao	Poaceae	Leaf	W	L	Cough R05, Asthma R96, Rheumatoid Arthritis L88, Influenza R80, Vaginal Discharge X14	0.61
NXYC121	*Oryza sativa* var.glutinosa Matsum	Nuo xi ken	Poaceae	Root	C	L	Dysuria U01	0.78
NXYC265	*Dactylicapnos scandens* (D. Don) Hutch	Ba shi niu niu	Papaveraceae	Root	W	L	Pain General/Multiple Sties A01, Hypertension K25, Abdominal Pain Epigastric D02	0.72
NXYC201	*Meconopsis racemosa* Maxim	Tiao shen	Papaveraceae	Root	W	L	Hepatitis D71, headache N01, Pain General/Multiple Sties A01	0.5
NXYC181	*Holboellia fargesii* Reaub	Yi zhi	Lardizabalaceae	Fruit, Stem	W	L	Pyelonephritis U70, Bronchitis R78, Chronic Enteritis/Ulcerative Colitis D94	0.61
NXYC248	*Sargentodoxa cuneata* (Oliv.) Rehd. et Wils	Ji xue teng	Lardizabalaceae	Root, Stem	W	L	Rheumatoid Arthritis L88, Appendicitis D88, Menstruation Irregular/Frequent X07	0.72
NXYC198	*Stephania epigaea* H. S. Lo	Wu mu ji du	Menispermaceae	Earthnut	W	L	Abdominal Pain Epigastric D02, Abdominal Pain D01, Rheumatoid Arthritis L88, Liver Disease NOS D97, Peptic Ulcer D86, Skin Disease Other S99, Parotitis R99	0.5
NXYC054	*Dysosma versipellis* (Hance) M.Cheng ex Ying	Ke ba guo lian	Berberidaceae	Root	W	L	Skin Disease Other S99, Bronchitis R78, Pain General/Multiple Sties A01, Abdominal Pain Epigastric D02, Neoplasm A21, Trauma A80	0.67
NXYC249	*Epimedium davidii* Franch	Zai piao qi	Berberidaceae	Flower	W	E	Rheumatoid Arthritis L88, lmpotence NOS Y07, Cough R05	0.94
NXYC045	*Mahonia bracteolata* Takeda	Xing dou han	Berberidaceae	Stem	W	L	Hepatitis D71, Burns/Scald S14, Chronic Enteritis/Ulcerative Colitis D94, Conjunctivitis F70, Diarrhea D11	0.83
NXYC263	*Aconitum brachypodum* Diels	Du pei	Ranunculaceae	Root	W	L	Pain General/Multiple Sties A01, Rheumatoid Arthritis L88, Teeth/Gum Disease D82, Skin Disease Other S99, Neoplasm A21	0.89
NXYC067	*Aconitum carmichaeli* Debx	Du la	Ranunculaceae	Root	W	L	Rheumatoid Arthritis L88, Abdominal Pain Epigastric D02, Pain General/Multiple Sties A01, Stroke K90	1
NXYC065	*Anemone vitifolia* Buch.-Ham. ex DC	Ban ji shu	Ranunculaceae	Root	W	L	Rheumatoid Arthritis L88, Worms/other Parasites D96	0.61
NXYC066	*Beesia calthaefolia* (Maxim.) Ulbr	Ju nu	Ranunculaceae	Whole plant	W	L	Rheumatoid Arthritis L88, Influenza R8, Boil Carbuncle S10	0.83
NXYC089	*Clematis argentilucida* (Levl. et Van.) W.T.Wang	Bai tou gong gong	Ranunculaceae	Stem	W	L	Post-partum Symptom/Complaint other W18, Dysuria U01, Urinary Infection U71, Pyelonephritis U70	0.94
NXYC001	*Clematis gouriana* Roxb. ex DC	Er ken li hai gou ze	Ranunculaceae	Stem	W	L	Rheumatoid Arthritis L88, Pain General/Multiple Sties A01, Dysuria U01	1
NXYC023	*Clematis montana* Buch.-Ham. ex DC	Ze diu ba	Ranunculaceae	Stem	W	L	Pyelonephritis U70, Urinary Infection U71, Urinary Calculi U95, Menstruation Irregular/Frequent X07, Breast/Lactation Symptom W19, Prostatitis Y73, Sleep Disturbance P06	0.94
NXYC268	*Clematis peterae* Hand.-Mazz	Mu tong	Ranunculaceae	Stem	W	L	Urinary Calculi U95, Urinary Infection U71, Pyelonephritis U70, Rheumatoid Arthritis L88, Sinusitis R75, Conjunctivitis F70, Menstruation Irregular/Frequent X07, Scabies S72	0.67
NXYC191	*Paeonia delavayi* Franch	Mu dan shou e	Paeoniaceae	Root	W	L	Hepatitis D71, Bleeding/Haemorrhage Nos A10, Pain General/Multiple Sties A01, Diarrhea D11	0.83
NXYC059	*Paeonia lactiflora* Pall	Mu dan shou	Paeoniaceae	Root	W	L	Menstruation Irregular/Frequent X07, Skin Disease Other S99, Pain General/Multiple Sties A01	0.89
NXYC103	*Liquidambar formosana* Hance	Lu lu tong	Altingiaceae	Fruit	W	E	Pyelonephritis U70, Rheumatoid Arthritis L88, Breast/Lactation Symptom W19, Menstruation Irregular/Frequent X07	1
NXYC037	*Bergenia purpurascens* (Hook.f.et Thom.) Engler	Guo chong ben	Saxifragaceae	Root	W	L	Influenza R80, Tuberculosis A70, Bleeding/Haemorrhage Nos A10, Rheumatoid Arthritis L88, Dyspepsia D07, Pain General/Multiple Sties A01, Pyelonephritis U70, Diarrhea D11	0.94
NXYC052	*Rodgersia sambucifolia* Hemsl	Yan tuo	Saxifragaceae	Root	W	L	Pain General/Multiple Sties A01, Menstruation Irregular/Frequent X07, Hematochezia D16, Rheumatoid Arthritis L88, Haematemesis D14, Influenza R80, Chronic Enteritis/Ulcerative Colitis D94	1
NXYC024	*Rhodiola fastigiata* (Hook. f. et Thoms.) S. H. Fu	Me ji xu	Crassulaceae	Root	W	L	Teeth/Gum Disease D82, Conjunctivitis F70, Heart Pain K01, Skin Disease Other S99	0.78
NXYC245	*Rhodiola crenulata* (Hook. f. et Thoms.) H. Ohba	Wu lu me ji xu	Crassulaceae	Stem, Root	W	L	Rheumatoid Arthritis L88, Pain General/Multiple Sties A01	0.61
NXYC150	*Rhodiola yunnanensis* Franch	Da du wu	Crassulaceae	Root	W	L	Rheumatoid Arthritis L88, Pain General/Multiple Sties A01, Breast Symptom Female other X21	0.67
NXYC176	*Cynomorium songaricum* Rupr	Suo yang	Cynomoriaceae	Stem	W	L	lmpotence NOS Y07, Constipation D12	0.72
NXYC272	*Tetrastigma hypoglaucum* Planch. ex Franch	Rou xue teng	Vitaceae	Root	W	E	Rheumatoid Arthritis L88, Pain General/Multiple Sties A01, Burns/Scald S14, Skin Disease Other S99	0.78
NXYC153	*Tetrastigma hypoglaucum* Planch.ex Franch	Tong si ban	Vitaceae	Root	W	L	Pain General/Multiple Sties A01, Rheumatoid Arthritis L88, Burns/Scald S14, Skin Disease Other S99	0.56
NXYC116	*Tetrastigma obtectum* (Wall.) Planch	A dong ming	Vitaceae	Root	W	L	Skin Disease Other S99, Rheumatoid Arthritis L88	0.78
NXYC061	*Campylotropis hirtella* (Franch.) Schindl	Ju nu gou	Fabaceae	Root	W	L	Abdominal Pain Epigastric D02, Burns/Scald S14, Menstruation Irregular/Frequent X07, Peptic Ulcer D86	0.78
NXYC165	*Crotalaria ferruginea* Grah. ex Benth	Zhu shi dou	Fabaceae	Whole plant	W	L	Pyelonephritis U70, Cystitis U71, Urinary Infection U71, Influenza R80, Vaginal Discharge X14	0.61
NXYC090	*Flemingia macrophylla* (Willd.) Merr	Qian jin ba	Fabaceae	Stem	W	L	Rheumatoid Arthritis L88, Impotence Y07, Peptic Ulcer D86, Influenza R80, Vaginal Discharge X14	0.78
NXYC096	*Gleditsia sinensis* Lam	Zhu ya zao	Fabaceae	Stem	W	L	Post-partum Symptom/Complaint other W18, Breast Symptom Female other X21, Question of Pregnancy W01, Skin Disease Other S99, Cough R05, Stroke K90	0.72
NXYC085	*Glycyrrhiza uralensis* Fisch	Fen cao	Fabaceae	Root	C	E	Poisoning by Medical Agent A84, Jaundice D13, Peptic Ulcer D86, Menstruation Irregular/Frequent X07, Laryngitis R77	1
NXYC020	*Pueraria lobata* (Willd.) Ohwi	Gan gan er	Fabaceae	Root	W, C	L	Influenza R80, Rash Localized S07, Infectious Disease A78, Heart Pain K01, Chronic Enteritis/Ulcerative Colitis D94	0.83
NXYC137	*Pueraria montana* var. thomsonii	Zi ge	Fabaceae	Flower	W	L	Rash Localized S07, Diabetes Mellitus T90, Hypertension K25	0.67
NXYC207	*Sophora flavescens* Aiton	Bai jing di gu	Fabaceae	Root	W	L	Skin Disease Other S99, Hemorrhoids K96, Vaginal Discharge X14, Syphilis Y70, Skin Disease Other S99, Vaginal Discharge X14, Diarrhea D11	0.89
NXYC228	*Sophora japonica* L	Huai mi	Fabaceae	Pistil	W, C	E, L	Hemorrhoids K96, Menstruation Irregular/Frequent X07, Diarrhea D11, Vaginal Discharge X14	0.78
NXYC164	*Spatholobus suberectus* Dunn	Ma lu hua	Fabaceae	Stem	W	L	Rheumatoid Arthritis L88, Menstruation Irregular/Frequent X07, lmpotence NOS Y07	0.94
NXYC025	*Polygala arillata* Buch.-Ham. ex D. Don	You mei hen ku	Polygalaceae	Root	W	L	Pyelonephritis U70, Pneumonia R81, Whooping Cough R71, Rheumatoid Arthritis L88, Tuberculosis A70, Pain General/Multiple Sties A01, Urinary Infection U71	0.78
NXYC098	*Polygala japonica* Houtt	Xiao yuan zhi	Polygalaceae	Whole plant	W	L	Hepatitis D71, Burns/Scald S14, Tonsillitis R76, Influenza R80, Abdominal Pain Epigastric D02, Rheumatoid Arthritis L88, Breast Symptom Female other X21, Trauma A80, Haematemesis D14, Urinary Calculi U95, Pneumonia R81	0.78
NXYC189	*Agrimonia pilosa* Ledeb	Guo ye	Rosaceae	Whole plant	W	L	Skin Disease Other S99, Abdominal Pain Epigastric D02, Abdominal Hernia other D91, Jaundice D13, Bleeding/Haemorrhage Nos A10, Vaginal Discharge X14, Pain General/Multiple Sties A01, Anemia B82, Influenza R80	1
NXYC131	*Prunus persica* L	Bu zhu e	Rosaceae	Bark	W	L	Burns/Scald S14, Boil Carbuncle S10	0.44
NXYC226	*Prunus armeniaca* L	Ai beng	Rosaceae	Seed	W	L	Cough R05, Constipation D12	0.89
NXYC104	*Eriobotrya japonica* (Thunb.) Lindl	Pi pa piao	Rosaceae	Leaf	W	L	Cough R05, Pyelonephritis U70, Cystitis U71, Bronchitis R78, Teeth/Gum Disease D82, Menstruation Irregular/Frequent X07, Urinary Infection U71	0.83
NXYC019	*Geum aleppicum* Jacq	Ji leng bu	Rosaceae	Whole plant	W, C	L	Hypertension K25, Menstruation Irregular/Frequent X07, Cough R05, Anemia B82	0.83
NXYC204	*Malus prunifolia* (Willd.) Borkh	Duo le lv	Rosaceae	Fruit	C	L	Diabetes Mellitus T90, Dyspepsia D07	0.78
NXYC184	*Potentilla fulgens* Wall. ex Hook	Yu lei yu mei guo ken	Rosaceae	Root	W	L	Dyspepsia D07, Burns/Scald S14, Bleeding/Haemorrhage Nos A10, Menstruation Irregular/Frequent X07, Vaginal Discharge X14	0.72
NXYC125	*Prinsepia utilis* Royle	Shu da gu de	Rosaceae	Stem	W	L	Breast Lump/Mass Female X19, Hemorrhoids K96, Rheumatoid Arthritis L88, Pain General/Multiple Sties A01, Dyspepsia D07, Anemia B82, Trauma A80, Skin Disease Other S99, Parotitis R99	0.94
NXYC170	*Pyracantha fortuneana* (Maxim.) Li	Huo ba guo	Rosaceae	Fruit	W	L	Hepatitis D71, Dyspepsia D07	0.67
NXYC202	*Rosa laevigata* Mich	Jin ying zi	Rosaceae	Fruit	W	L	Pyelonephritis U70, Menstruation Irregular/Frequent X07, Pain General/Multiple Sties A01	0.67
NXYC147	*Rosa omeoensis* Rolfe	Zai yang guo	Rosaceae	Fruit	W	L	Worms/other Parasites D96, Bleeding/Haemorrhage Nos A10, Chronic Enteritis/Ulcerative Colitis D94, Vaginal Discharge X14	0.44
NXYC190	*Rubus delavayi* Franch	Xiao ci guo	Rosaceae	Whole plant	W	L	Skin Disease Other S99, Tonsillitis R76, Worms/other Parasites D96, Vaginal Discharge X14	0.39
NXYC231	*Sanguisorba filiformis* (Hook.f.) Hand.-Mazz	Wu mu na ba	Rosaceae	Root	W	L	Menstruation Irregular/Frequent X07, Infertility Female W15	0.56
NXYC183	*Sanguisorba officinalis* L	Di yu	Rosaceae	Root	W	L	Dyspepsia D07, Hemorrhoids K96, Menstruation Irregular/Frequent X07, Bleeding/Haemorrhage Nos A10	0.83
NXYC118	*Morus alba* L	Qi zi	Moraceae	Stem	W	L	Deafness H86, Dizziness N17	0.94
NXYC197	*Laportea cuspidate* (Wedd.) Friis	Me pei xu ken	Urticaceae	Root	W	L	Pyelonephritis U70, Liver Disease NOS D97, Rheumatoid Arthritis L88	0.61
NXYC032	*Alnus nepalensis* D. Don	Ji nong e	Betulaceae	Bark	W	L	Poisoning by Medical Agent A84, Rheumatoid Arthritis L88, Pain General/Multiple Sties A01, Diarrhea D11	0.78
NXYC155	*Benincasa hispida* (Thunb.) Cogn	Dong gua zi	Cucurbitaceae	Seed	W	L	Diabetes Mellitus T90, Skin Disease Other S99, Influenza R80, Vaginal Discharge X14, Tetanus N72	0.78
NXYC091	*Euonymus alatus* (Thunb.) Sieb	Gui jian yu	Celastraceae	Stem	W	L	Worms/other Parasites D96, Menstruation Irregular/Frequent X07, Rheumatoid Arthritis L88	0.78
NXYC099	*Euonymus grandiflorus* Wall	Shi xiao dou	Celastraceae	Bark	W	L	Menstruation Irregular/Frequent X07, Rheumatoid Arthritis L88, Pain General/Multiple Sties A01, Diarrhea D11	0.94
NXYC077	*Oxalis corniculata* L	Leng gan se ji	Oxalidaceae	Whole plant	W	L	Jaundice D13, Hemorrhoids K96, Menstruation Irregular/Frequent X07, Influenza R80, Vaginal Discharge X14	0.67
NXYC038	*Hypericum hookerianum* Wight &Arnott	Ni mei hei tu ba	Hypericaceae	Whole plant	W	L	Hepatitis D71, Pyelonephritis U70, Chronic Enteritis/Ulcerative Colitis D94. Cystitis U71, Pruritus S02, Menstruation Irregular/Frequent X07, Diarrhea D11	0.78
NXYC205	*Euphorbia jolkinii* Boiss	Qiong du de	Euphorbiaceae	Root	W	L	Scabies S72, Pain General/Multiple Sties A01, Neoplasm A21	0.67
NXYC029	*Geranium strictipes* R.Knuth	Qie san che e	Geraniaceae	Root	W	L	Dyspepsia D07, Chronic Enteritis/Ulcerative Colitis D94, Hematochezia D16, Diarrhea D11	0.89
NXYC252	*Geranium wallichianum* D. Don ex Sweet	Qie san che e pei	Geraniaceae	Root	W	L	Abdominal Pain Epigastric D02, Dyspepsia D07, Diarrhea D11, Rheumatoid Arthritis L88	0.89
NXYC133	*Dobinea delavayi* Baill	Jiu zi bu li mu	Anacardiaceae	Root	W	L	Breast Lump/Mass Female X19, Pain General/Multiple Sties A01, Skin Disease Other S99, Parotitis R99	0.72
NXYC106	*Rhus chinensis* Mill	Mei piao hua	Anacardiaceae	Others	W	L	Cough R05, Diabetes Mellitus T90, Hematochezia D16	0.94
NXYC277	*Rhus potaninii* Maxim	Mei piao lv	Anacardiaceae	Others	W	L	Hemorrhoids K96, Menstruation Irregular/Frequent X07, Cough R05, Burns/Scald S14, Diarrhea D11, Pain General/Multiple Sties A01, Skin Disease Other S99	0.83
NXYC074	*Boenninghausenia sessilicarpa* Levl	She pi cao	Rutaceae	Whole plant	W	L	Tonsillitis R76, Dysuria U01, Skin Disease Other S99, Abdominal Pain Epigastric D02, Influenza R80, Malaria A73, Bleeding/Haemorrhage Nos A10, Parotitis R99, Diarrhea D11	0.78
NXYC087	*Melicope pteleifolia* (Champion ex Bentham) T. G. Hartley	Xiao huang san	Rutaceae	Stem	W	E	Hepatitis D71, Rheumatoid Arthritis L88, Pain General/Multiple Sties A01, Upper Respiratory Infection R74, Skin Disease Other S99, Trauma A80, Jaundice D13, Pneumonia R81	0.72
NXYC270	*Toddalia asiatica* (L.) Lam	Fei long zhang xue	Rutaceae	Root, Stem	W	L	Rheumatoid Arthritis L88, Pain General/Multiple Sties A01, Menstruation Irregular/Frequent X07, Abdominal Pain Epigastric D02, Haematemesis D14, Skin Disease Other S99	0.61
NXYC214	*Zanthoxylum bungeanum* Maxim	Zui cai	Rutaceae	Others	W	L	Other Parasites D96, Dermatitis S87, Abdominal Pain D01	0.67
NXYC003	*Melia azedarach* L	Da lv lv	Meliaceae	Fruit	W	L	Menstruation Irregular/Frequent X07, Worms/other Parasites D96, Abdominal Hernia other D91, Boil Carbuncle S10, Pain General/Multiple Sties A01	0.89
NXYC227	*Isatis tinctoria* L	Ban lan gen	Brassicaceae	Root	W, C	E, L	Hepatitis D71, Influenza R80, Skin Disease Other S99, Tuberculosis A70, Parotitis R99	0.94
NXYC174	*Balanophora involucrata* Hook. f	Di chang	Balanophoraceae	Whole plant	W	L	lmpotence NOS Y07, Menstruation Irregular/Frequent X07, Pain General/Multiple Sties A01, Influenza R80, Hepatitis D71	0.67
NXYC171	*Arceuthobium pini* Hawksw. et Wiens	Tuo xiu	Santalaceae	Stem	W	L	Rheumatoid Arthritis L88, Diarrhea D11, Pain General/Multiple Sties A01	0.56
NXYC081	*Taxillus delavayi* (Van Tiegh.) Danser	Ge zi chang	Loranthaceae	Whole plant	W	L	Abortion Spontaneous W82, Rheumatoid Arthritis L88	0.83
NXYC208	*Fagopyrum dibotrys* (D. Don) Hara	Ruo a kao ken	Polygonaceae	Root	W	L	Dyspepsia D07, Menstruation Irregular/Frequent X07, Abdominal Pain Epigastric D02, Post-partum Symptom/Complaint other W18	0.72
NXYC148	*Polygonum amplexicaule* D. Don	Ai ji ken	Polygonaceae	Root	W	L	Pain General/Multiple Sties A01, Rheumatoid Arthritis L88, Abdominal Pain D01, Diarrhea D11	0.61
NXYC129	*Polygonum aviculare* L	Bian xu	Polygonaceae	Whole plant	W	L	Skin Disease Other S99, Jaundice D13, Pyelonephritis U70, Vaginal Discharge X14	0.78
NXYC039	*Polygonum cuspidatum* Sieb. et Zucc	La me tu	Polygonaceae	Root	W	L	Rheumatoid Arthritis L88, Pain General/Multiple Sties A01, Jaundice D13, Hepatitis D71, Malignant Neoplasms Stomach D74, Post-partum Symptom/Complaint other W18, Menstruation Irregular/Frequent X07, Burns/Scald S14	1
NXYC040	*Polygonum multiflorum* Thunb	Tuo san qi	Polygonaceae	Root	W	L	Hepatitis D71, Pruritus S02, Benign Neoplasm Female Genital X80, Lipid Disorder T93, Constipation D12	1
NXYC167	*Polygonum paleaceum* Wall	Jie ken	Polygonaceae	Root	W	L	Bleeding/Haemorrhage Nos A10, Menstruation Irregular/Frequent X07, Pain General/Multiple Sties A01, Burns/Scald S14, Cataract F92, Jaundice D13, Neoplasm A21, Chronic Enteritis/Ulcerative Colitis D94, Skin Disease Other S99, Vaginal Discharge X14	0.72
NXYC146	*Rheum delavayi* Franch	Lu zei ken	Polygonaceae	Root	W	L	Rheumatoid Arthritis L88, Pain General/Multiple Sties A01, Hematochezia D16, Influenza R80	0.5
NXYC060	*Rheum likiangense* Sam	Xi pu gao nu na	Polygonaceae	Root	W	L	Menstruation Irregular/Frequent X07, Constipation D12, Pain General/Multiple Sties A01, Parotitis R99, Diarrhea D11	0.78
NXYC022	*Rheum palmatum* L	Hua zeng de	Polygonaceae	Root	W	L	Constipation D12, Post-partum Symptom/Complaint other W18, Teeth/Gum Disease D82, Menstruation Irregular/Frequent X07, Dyspepsia D07, Jaundice D13, Pain General/Multiple Sties A01, Vomiting D10, Haematemesis D14, Liver Disease NOS D97, Burns/Scald S14	1
NXYC044	*Rumex nepalensis* Spreng	Hua leng hua zeng ke	Polygonaceae	Root	W	L	Hemorrhoids K96, Jaundice D13, Chronic Enteritis/Ulcerative Colitis D94, Pain General/Multiple Sties A01, Bleeding/Haemorrhage Nos A10, Burns/Scald S14, Skin Disease Other S99, Teeth/Gum Disease D82, Diarrhea D11, Perianal Abscess D95, Parotitis R99	0.94
NXYC216	*Arenaria serpyllifolia* L	Sao zhui	Caryophyllaceae	Whole plant	W	L	Hepatitis D71, Tuberculosis A70, Tonsillitis R76, Conjunctivitis F70, Rheumatoid Arthritis L88, Sinusitis R75, Deafness H86, Trauma A80	0.67
NXYC213	*Psammosilene tunicoides* W. C. Wu et C. Y. Wu	Di yu	Caryophyllaceae	Root	W	L	Rheumatoid Arthritis L88, Pain General/Multiple Sties A01, Worms/other Parasites D96, Abdominal Pain Epigastric D02, Trauma A80	0.56
NXYC254	*Silene viscidula* Franch	Wu zhong ke	Caryophyllaceae	Root	W	L	Boil/Carbuncle S10, Menstruation Irregular/Frequent X07, Pain General/Multiple Sties A01	0.5
NXYC158	*Achyranthes bidentata* Bl	Ji e gu zi zi	Amaranthaceae	Root	W	L	Hypertension K25, Rheumatoid Arthritis L88, Menstruation Irregular/Frequent X07,	1
NXYC031	*Phytolacca acinosa* Roxb	A you ke hu pai	Phytolaccaceae	Root	W	L	Pyelonephritis U70, Skin Disease Other S99, Liver Disease NOS D97	1
NXYC021	*Mirabilis jalapa* L	Zi mo li	Nyctaginaceae	Root	W	L	Tonsillitis R76, Diabetes Mellitus T90, Prostatitis Y73, Menstruation Irregular/Frequent X07, Urinary Infection U71, Haematemesis D14	0.78
NXYC275	*Alangium chinense* (Lour.) Harms	Huo guo piao zei	Cornaceae	Root, Stem	W	E	Rheumatoid Arthritis L88, Pain General/Multiple Sties A01, Dystocia, Jaundice D13	0.72
NXYC185	*Lysimachia christinae* Hance	Hai ken ba	Primulaceae	Whole plant	W	L	Urinary Infection U71, Breast Symptom Female other X21, prostatic hypertrophy Y85, Pyelonephritis U70, Jaundice D13, Hemorrhoids K96, Pain General/Multiple Sties A01, Tuberculosis A70, Urinary Calculi U95	0.94
NXYC083	*Lysimachia congestiflora* Hensl	Hai ken ba	Primulaceae	Whole plant	W	L	Pyelonephritis U70, Skin Disease Other S99, Urinary Calculi U95, Influenza R80	1
NXYC212	*Primula forrestii* Balf. F	Ai ji ceng	Primulaceae	Root	W	L	Pain General/Multiple Sties A01, Rheumatoid Arthritis L88	0.61
NXYC130	*Agapetes mannii* Hemsl	Shu luo bo	Ericaceae	Root	W	L	Hepatitis D71, Menstruation Irregular/Frequent X07, Pain General/Multiple Sties A01	0.5
NXYC068	*Cassiope selaginoides* Hook.f. et Thoms	Tuo bei lei	Ericaceae	Whole plant	W	L	Rheumatoid Arthritis L88, Dyspepsia D07, Dizziness N17, Neurasthenia P78	0.67
NXYC114	*Gaultheria forrestii* Diels	Ke ha lu ge	Ericaceae	Whole plant	W	L	Cough R05, Rheumatoid Arthritis L88, Tuberculosis A70	0.72
NXYC229	*Pyrola atropurpurea* Franch	Jiong gu lai	Ericaceae	Flower	W	L	Dermatitis Allergic S88, Bronchitis R78, Rheumatoid Arthritis L88, Tuberculosis A70	0.83
NXYC154	*Pyrola forrestiana* H. Andres	Guang huang cao	Ericaceae	Whole plant	W	L	Tuberculosis A70, Influenza R80, Diarrhea D11	0.83
NXYC132	*Rhododendron delavayi* Franch	Ma ying hua	Ericaceae	Flower	W	L	Post-partum Symptom/Complaint other W18, Menstruation Irregular/Frequent X07, Bleeding/Haemorrhage Nos A10, Dysuria U01, Skin Disease Other S99, Diarrhea D11	0.56
NXYC035	*Eucommia ulmoides* Oliv	E mian kuo	Eucommiaceae	Bark, Seed	W, C	L	Rheumatoid Arthritis L88, Abortion Spontaneous W82, Hypertension K25, Vaginal Discharge X14, Pain General/Multiple Sties A01	0.94
NXYC234	*Galium elegans* Wall	Niu zhou ken xu	Rubiaceae	Root	W	L	Carbuncle S10, Urinary Infection U71, Pain General/Multiple Sties A01, Trauma A80, Vaginal Discharge X14	0.89
NXYC259	*Hedyotis diffusa* Willd	Bai hua she she cao	Rubiaceae	Flower	W	L	Neoplasm A21, Appendicitis D88, Pyelonephritis U70, Urinary Infection U71, Bronchitis R78, Breast Symptom Female other X21, Hepatitis D71, Tonsillitis R76, Skin Disease Other S99, Trauma A80	0.83
NXYC144	*Rubia schumanniana* Pritz	San xing che e	Rubiaceae	Root	W	L	Menstruation Irregular/Frequent X07, Bleeding/Haemorrhage Nos A10, Rheumatoid Arthritis L88, Skin Disease Other S99	0.72
NXYC209	*Rubia yunnanensis* Diels	Niu zhou piao ba ken	Rubiaceae	Whole plant	W	L	Menstruation Irregular/Frequent X07, Hepatitis D71, Bleeding/Haemorrhage Nos A10, Rheumatoid Arthritis L88	0.61
NXYC273	*Uncaria macrophylla* Wall. in Roxb	Shuang gou teng	Rubiaceae	Stem, Root	W	L	headache N01, Epilepsy N88, Rheumatoid Arthritis L88	0.89
NXYC196	*Uncaria sinensis* (Oliv.) Havil	Gou teng	Rubiaceae	Stem	W	E	Vertigo/Dizziness N17, headache N01, Epilepsy N88, Rheumatoid Arthritis L88	0.94
NXYC173	*Gentiana crassicaulis* Duthie ex Burk	Luo bo qin jiao	Gentianaceae	Root	W, C	L	Dysuria U01, Abdominal Pain Epigastric D02	0.67
NXYC200	*Gentiana rhodantha* Franch. ex Hemsl	Ji ba ka	Gentianaceae	Whole plant	W	L	Jaundice D13, Influenza R80, Tuberculosis A70, Burns/Scald S14, Asthma R96, Skin Disease Other S99, Vaginal Discharge X14	0.67
NXYC017	*Gentiana rigescens* Franch.ex Hemsl	Ji kao	Gentianaceae	Whole plant	W	L	Conjunctivitis F70,Malaria A73, Hemorrhoids K96, Hepatitis D71, Rash Localized S07, Tonsillitis R76, Breast Symptom Female other X21, Epilepsy N88, Pneumonia R81, Diarrhea D11	0.94
NXYC221	*Gentiana yunnanensis* Franch	Ji ka	Gentianaceae	Whole plant	W	L	Hepatitis D71, Infectious Disease A78	0.44
NXYC018	*Swertia punicea* Hemsl	Ji kao	Gentianaceae	Whole plant	W	L	Teeth/Gum Disease D82	0.89
NXYC149	*Veratrilla baillonii* Franch	Te gu che er	Gentianaceae	Root	W	L	Breast Symptom Female other X21, Hepatitis D71, Chronic Enteritis/Ulcerative Colitis D94, Influenza R80, Pain General/Multiple Sties A01, Pyelonephritis U70, Skin Disease Other S99, Poisoning by Medical Agent A84	0.67
NXYC262	*Strychnos nux-vomica* L	Ku shi	Loganiaceae	Seed	W	L	Heart Pain K01, Laryngitis R77	0.78
NXYC028	*Cynanchum otophyllum* Schneid	La gan zi yi	Apocynaceae	Root	W	L	Rash Localized S07, Epilepsy N88, Trauma A80, Rheumatoid Arthritis L88, Dyspepsia D07	1
NXYC276	*Marsdenia tenacissima* (Roxb.) Wight	Tong guang san	Apocynaceae	Stem	W	E	Dysuria U01, Breast/Lactation Symptom W19, Abdominal Pain Epigastric D02	0.72
NXYC271	*Periploca forrestii* Schltr	Nian er na	Apocynaceae	Stem, Root	W	L	Rheumatoid Arthritis L88, Pain General/Multiple Sties A01, Breast Symptom Female other X21, Skin Disease Other S99	0.67
NXYC260	*Cynoglossum amabile* Stapf & J. R. Drumm	Dao ti hu	Boraginaceae	Flower	W	L	Cystitis U71, Urethritis U72, Jaundice D13, Skin Disease Other S99, Pyelonephritis U70, Hematochezia D16, Laryngitis R77	0.61
NXYC005	*Onosma paniculatum* Bur.et Franch	Lao rao ken	Boraginaceae	Root	W	L	Rash Localized S07, Burns/Scald S14	0.94
NXYC251	*Cuscuta chinensis* Lam	Ci mian lv	Convolvulaceae	Seed	W	L	lmpotence NOS Y07, Abortion Spontaneous W82, Dizziness N17	0.83
NXYC080	*Cuscuta japonica* Choisy		Convolvulaceae	Stem	W	L	Infertility Female W15, Impotence Y07	0.72
NXYC139	*Dichondra repens* Forst	He bao cao	Convolvulaceae	Whole plant	W	L	Hepatitis D71, Pyelonephritis U70, Breast Lump/Mass Female X19, Urinary Infection U71, Cholecystitis D98, Influenza R80, Tonsillitis R76, Skin Disease Other S99	0.56
NXYC163	*Anisodus acutangulus* C. Y. Wu et C. Chen	Du pei ken bei	Solanaceae	Root	W	L	Pain General/Multiple Sties A01, Rheumatoid Arthritis L88, Menstruation Irregular/Frequent X07, Chronic Enteritis/Ulcerative Colitis D94, Meningitis N71	0.78
NXYC136	*Solanum lyratum* Thunb	Bai ying	Solanaceae	Whole plant	W	L	Hepatitis D71, Pyelonephritis U70, Influenza R80, Rheumatoid Arthritis L88, Vaginal Discharge X14	0.72
NXYC111	*Solanum nigrum* L	Ruo la ze	Solanaceae	Stem, Leaf	W	L	Influenza R80, Urinary Calculi U95, Pyelonephritis U70, Neoplasm A21, Tonsillitis R76, Urinary Infection U71, Breast Symptom Female other X21, Trauma A80, Skin Disease Other S99, Teeth/Gum Disease D82	0.78
NXYC274	*Corallodiscus flabellatus* (Craib) B. L. Burtt	Shi hua	Gesneriaceae	Flower	W	L	Menstruation Irregular/Frequent X07, Skin Disease Other S99, Infertility Female W15, lmpotence NOS Y07, Abdominal Pain Epigastric D02, Pain General/Multiple Sties A01, Palpitation K04, Poisoning by Medical Agent A84, Vaginal Discharge X14	0.67
NXYC076	*Hemiphragma heterophyllum* Wall	A you jian da ken	Plantaginaceae	Whole plant	W	L	Rheumatoid Arthritis L88, Menstruation Irregular/Frequent X07, Pain General/Multiple Sties A01, Neoplasm A21	0.89
NXYC079	*Plantago asiatica* L	Bo mei ji gu da	Plantaginaceae	Whole plant	W	L	Dysuria U01, Cataract F92, Skin Disease Other S99, Influenza R80	0.89
NXYC113	*Veronica anagallis-aquatica* L	Shui suo cao	Plantaginaceae	Whole plant	W	L	Bleeding/Haemorrhage Nos A10, Cough R05, Pain General/Multiple Sties A01, Rheumatoid Arthritis L88, Menstruation Irregular/Frequent X07, Skin Disease Other S99	0.78
NXYC160	*Verbascum thapsus* L	Da mao ye	Scrophulariaceae	Whole plant	W	L	Cystitis U71, Influenza R80, Urethritis U72, Urticaria S98, Influenza R80, Chronic Enteritis/Ulcerative Colitis D94, Pain General/Multiple Sties A01, Skin Disease Other S99	0.67
NXYC072	*Verbena officinalis* L	Ruan miu bi	Verbenaceae	Whole plant	W	L	Hepatitis D71, Influenza R80, Laryngitis R77, Pyelonephritis U70, Pain in Testis/Scrotum Y02, Skin Disease Other S99, Teeth/Gum Disease D82, Chronic Enteritis/Ulcerative Colitis D94, Worms/other Parasites D96, Malaria A73, Menstruation Irregular/Frequent X07	0.94
NXYC161	*Ajuga forrestii* Diels	Jin gu cao	Lamiaceae	Whole plant	W	L	Breast Symptom Female other X21, Jaundice D13, Carbuncle S16, Influenza R80, Urinary Calculi U95, Diarrhea D11, Otitis Media H74	0.61
NXYC123	*Clerodendranthus spicatus* (Thunb.) C. Y. Wu. ex H. W. Li	Ya nu miao	Lamiaceae	Whole plant	W	E	Cystitis U71, Urinary Calculi U95, Cholelithiasis D98, Pyelonephritis U70	0.78
NXYC063	*Elsholtzia blanda* (Benth.) Benth	Jia su	Lamiaceae	Whole plant	W	L	Influenza R80, Rash Localized S07	0.78
NXYC152	*Isodon yuennanensis* (Hand.-Mazz.) H. Hara	Beng mei ju ken	Lamiaceae	Root	W	L	Menstruation Irregular/Frequent X07, Pain General/Multiple Sties A01, Abdominal Pain Epigastric D02	0.67
NXYC071	*Leonurus artemisia* (Lour.) S. Y. Hu	Ba pei mei che	Lamiaceae	Whole plant	W	L	Menstruation Irregular/Frequent X07, Question of Pregnancy W01, Post-partum Symptom/Complaint other W18, Skin Disease Other S99	0.89
NXYC180	*Origanum vulgare* L	Yu lu a zhi	Lamiaceae	Whole plant	W	L	Influenza R80, Chronic Enteritis/Ulcerative Colitis D94	1
NXYC217	*Phlomis betonicoides* Diels	Xin shen	Lamiaceae	Root	W	L	Dyspepsia D07, Bronchitis R78	0.44
NXYC058	*Prunella hispida* Benth	Rong bu shi	Lamiaceae	Whole plant	W	L	Neoplasm A21, Breast Lump/Mass Female X19, Tuberculosis A70, Hepatitis D71, Cataract F92, Pain General/Multiple Sties A01, Influenza R80, Goiter T81, Stroke K90, Parotitis R99, Vaginal Discharge X14	1
NXYC168	*Salvia flava*	Bai qi piao	Lamiaceae	Root	W	L	Menstruation Irregular/Frequent X07, Sleep Disturbance P06, Pain General/Multiple Sties A01, Teeth/Gum Disease D82	0.67
NXYC238	*Salvia przewalskii* Maxim	Fu ken man	Lamiaceae	Root	C	E	Abdominal Hernia other D91, Sleep Disturbance P06, Pain General/Multiple Sties A01, Menstruation Irregular/Frequent X07, Heart Pain K01, Skin Disease Other S99	0.67
NXYC126	*Salvia trijuga* Diels	Xiao hong shen	Lamiaceae	Root	W	L	Menstruation Irregular/Frequent X07, Liver Disease NOS D97, Impotence Y07	0.72
NXYC108	*Salvia yunnanensis* C. H. Wright	Bai qi piao	Lamiaceae	Root	W	L	Menstruation Irregular/Frequent X07, Breast Lump/Mass Female X19, Post-partum Symptom/Complaint other W18, Skin Disease Other S99, Pain General/Multiple Sties A01, Liver Disease NOS D97, Hepatitis D71	0.94
NXYC062	*Scutellaria amoena* C. H. Wright	Ken shi ba hen	Lamiaceae	Root	W	L	Cough R05, Jaundice D13, Bleeding/Haemorrhage Nos A10, Diarrhea D11, Abortion Spontaneous W82	0.89
NXYC073	*Scutellaria barbata* D. Don	Shi gong che e	Lamiaceae	Stem, Leaf	W	L	Urinary Infection U71, Hepatitis D71, Neoplasm A21, Breast Symptom Female other X21, Liver Disease NOS D97, Cystitis U71, Bleeding/Haemorrhage Nos A10, Jaundice D13, Trauma A80, Tuberculosis A70	0.89
NXYC253	*Boschniakia himalaica* Hook. f. et Thoms	Qian jin zhui	Orobanchaceae	Flower, Root	W	L	lmpotence NOS Y07, Other Parasites D96, Abdominal Pain D01, Abdominal Hernia other D91, Rheumatoid Arthritis L88, Pain General/Multiple Sties A01, Cough R05, Laryngitis R77, Poisoning by Medical Agent A84, Parotitis R99	0.72
NXYC240	*Cistanche deserticola* Y. C. Ma	Rou cong rong	Orobanchaceae	Stem	C	E	lmpotence NOS Y07, Infertility Female W15, Constipation D12, Abdominal Pain Epigastric D02, Vaginal Discharge X14	0.94
NXYC092	*Adenophora khasiana* (Hook. f. et Thomson) Oliv. ex Collett et Hemsl	Ba hen shi shua ken	Campanulaceae	Root	W	L	Bronchitis R78, Whooping Cough R71, Diabetes Mellitus T90, Skin Disease Other S99	0.89
NXYC026	*Codonopsis subglobosa* W. W. Smith	Xu dang	Campanulaceae	Root	W	L	Diabetes Mellitus T90, Heart Pain K01, Dyspepsia D07, Diarrhea D11	0.94
NXYC215	*Platycodon grandiflorum* (Jacq.) A. DC	Ji gen ken	Campanulaceae	Root	W	L	Bronchitis R78, Tuberculosis A70	0.78
NXYC006	*Aucklandia costus* Falc	Sa dui ken bi	Asteraceae	Root	C	L	Vomiting D10, Diarrhea D11, Dyspepsia D07	0.89
NXYC186	*Ainsliaea latifolia* (D. Don) Sch.-Bip	Mao jiao wei ling xian	Asteraceae	Root	W	L	Abdominal Pain D01, Pain General/Multiple Sties A01, Conjunctivitis F70	0.56
NXYC243	*Ainsliaea pertyoides* var. albo-tomentosa	Piao tai ba	Asteraceae	Root	W	L	Pain General/Multiple Sties A01, Rheumatoid Arthritis L88, Post-partum Symptom/Complaint other W18, Dermatitis Allergic S88, Menstruation Irregular/Frequent X07	0.67
NXYC042	*Arctium lappa* L	E mei la ba	Asteraceae	Root	W	L	Influenza R80, Boil Carbuncle S10, Skin Disease Other S99	0.89
NXYC069	*Artemisia lancea* Van	Gei shi gong shen a	Asteraceae	Whole plant	W	L	Influenza R80, Hepatitis D71, Malaria A73, Trauma A80, Skin Disease Other S99	0.89
NXYC166	*Artemisia sieversiana* Ehrhart	Beng ka	Asteraceae	Whole plant	W	L	Rheumatoid Arthritis L88, Pain General/Multiple Sties A01, Skin Disease Other S99, Bleeding/Haemorrhage Nos A10, Influenza R80	0.83
NXYC016	*Aster jeffreyanus* Diels	Lu mi	Asteraceae	Root	W	L	Boil Carbuncle S10, Cough R05, Infectious Disease A78	1
NXYC138	*Atractylodes macrocephala* Koidz	Chao bai zhu	Asteraceae	Root	W	L	Dysuria U01, Jaundice D13, Dyspepsia D07, Abortion Spontaneous W82	0.83
NXYC122	*Bidens pilosa* L	Yi bao zhen	Asteraceae	Whole plant	W	L	Malaria A73, Hypertension K25, Appendicitis D88, Jaundice D13, Influenza R80, Dyspepsia D07, Menstruation Irregular/Frequent X07, Rheumatoid Arthritis L88, Neoplasm A21, Skin Disease Other S99	0.67
NXYC086	*Carthamus tinctorius* L	Ci hong hua	Asteraceae	Flower	C	L	Palpitation K04, Liver Disease NOS D97, Anemia B82, Pain General/Multiple Sties A01, Urinary Calculi U95, Menstruation Irregular/Frequent X07, Rheumatoid Arthritis L88, Infertility Female W15, Skin Disease Other S99	1
NXYC078	*Cirsium japonicum* Fisch. ex DC	Ba qi	Asteraceae	Root	W	L	Peptic Ulcer D86, Pain General/Multiple Sties A01, Burns/Scald S14, Skin Disease Other S99, Hypertension K25, Vaginal Discharge X14	1
NXYC014	*Crepis phoenix* Dunn	Ke shua ri	Asteraceae	Root	W	L	Hepatitis D71, Breast/Lactation Symptom W19, Pruritus S02	0.83
NXYC105	*Dendranthema morifolium* (Ramat.) Tzvel	Ju hua	Asteraceae	Flower	W	L	Influenza R80, Deafness H86, Conjunctivitis F70, Hypertension K25, Skin Disease Other S99	0.94
NXYC075	*Dichrocephala auriculate* (Thunb.) Druce	Ni mei ge ru ji	Asteraceae	Whole plant	W	L	Laryngitis R77, Skin Disease Other S99, Pain General/Multiple Sties A01	0.94
NXYC188	*Dolomiaea berardioidea* (Franch.) Shih	Guo bai bai	Asteraceae	Root	W	L	Dyspepsia D07, Bronchitis R78, Chronic Enteritis/Ulcerative Colitis D94	0.94
NXYC195	*Eclipta prostrata* L	Ni men da ba xiao	Asteraceae	Whole plant	W	L	Hepatitis D71, Bleeding/Haemorrhage Nos A10, Skin Disease Other S99, Diarrhea D11, Tetanus N72	0.89
NXYC143	*Erigeron breviscapus* (Vant.) Hand.-Mazz	Dong ju	Asteraceae	Whole plant	W	L	Rheumatoid Arthritis L88, Teeth/Gum Disease D82, Influenza R80, Diarrhea D11	0.56
NXYC070	*Eupatorium fortunei* Turcz	Peng lai	Asteraceae	Whole plant	W	L	Influenza R80, Menstruation Irregular/Frequent X07, Diabetes Mellitus T90, Pain General/Multiple Sties A01	0.89
NXYC057	*Gerbera delavayi* Franch	Ju ben	Asteraceae	Whole plant	W	L	Dyspepsia D07, Worms/other Parasites D96, Diarrhea D11	0.72
NXYC187	*Gynura japonica* (Thunb.) Juel	Ze lan shi	Asteraceae	Root	W	L	Pain General/Multiple Sties A01, Post-partum Symptom/Complaint other W18, Breast Symptom Female other X21, Tonsillitis R76	0.78
NXYC222	*Hippolytia delavayi* (Franch. ex W. W. Smith) Shih	Beng lei ru	Asteraceae	Root	W	L	Tuberculosis A70, Cough R05, Bronchitis R78	0.5
NXYC257	*Inula nervosa* Wall. ex Hook.f	Ju wei ling	Asteraceae	Root	W	L	Abdominal Pain Epigastric D02, Dyspepsia D07, Dizziness N17	0.44
NXYC194	*Laggera crispata* (Vahl) Hepper et J	Liu leng ju	Asteraceae	Whole plant	W	L	Influenza R80, Teeth/Gum Disease D82, Tuberculosis A70, Urinary Infection U71, Trauma A80, Skin Disease Other S99	0.72
NXYC056	*Petasites tricholobus* Franch	Di hu lu	Asteraceae	Whole plant	W	L	Pain General/Multiple Sties A01, Skin Disease Other S99, Constipation D12	0.61
NXYC258	*Saussurea deltoidea* (DC.) Sch.-Bip	Mao ye wei ling xian	Asteraceae	Root	W	L	Rheumatoid Arthritis L88, Abdominal Pain Epigastric D02, Diarrhea D11, Vaginal Discharge X14	0.61
NXYC178	*Saussurea leucoma* Diels	Ji se yu lei	Asteraceae	Whole plant	W	L	Menstruation Irregular/Frequent X07, Tuberculosis A70, Pain General/Multiple Sties A01	0.94
NXYC211	*Saussurea romuleifolia* Franch	ri miu ru	Asteraceae	Whole plant	W	L	Rheumatoid Arthritis L88, Pain General/Multiple Sties A01, Trauma A80	0.61
NXYC169	*Senecio scandens* Buch.-Ham	Ba shi ba	Asteraceae	Whole plant	W	L	Influenza R80, Vaginal Discharge X14, Boil Carbuncle S10, Hepatitis D71, Cholecystitis D98, Cataract F92, Burns/Scald S14, Meningitis N71	0.83
NXYC046	*Taraxacum mongolicum* Hand.-Mazz	Bo xiao mi	Asteraceae	Root, Whole plant	W, C	L	Upper Respiratory Infection R74, Tonsillitis R76, Conjunctivitis F70, Breast Symptom Female other X21, Chronic Enteritis/Ulcerative Colitis D94, Appendicitis D88, Hepatitis D71, Urinary Infection U71, Diarrhea D11, Parotitis R99	1
NXYC244	*Wedelia urticifolia* (Bl.) DC	Di xue shen	Asteraceae	Root	W	L	Pain General/Multiple Sties A01	0.72
NXYC182	*Xanthium sibiricum* Patrin ex Widder	Fu gu du	Asteraceae	Fruit	W	L	Rheumatoid Arthritis L88, Malaria A73, Sinusitis R75, Rash Localized S07, Trauma A80, Pruritus S02	1
NXYC050	*Sambucus williamsii* Hance	Wo zhu ze	Adoxaceae	Bark	W	L	Pain General/Multiple Sties A01, Rheumatoid Arthritis L88, Pyelonephritis U70	0.94
NXYC030	*Dipsacus asper* Wall. ex Henry	Qi ke du lu	Caprifoliaceae	Root	W, C	L	Rheumatoid Arthritis L88, Abortion Spontaneous W82, Breast/Lactation Symptom W19, Infertility Female W15, Breast Lump/Mass Female X19	1
NXYC112	*Leycesteria formosa* Wall	Ji wei wei	Caprifoliaceae	Stem, Leaf	W	L	Rheumatoid Arthritis L88, Jaundice D13, Menstruation Irregular/Frequent X07, Cystitis U71, Hemorrhoids K96, Dyspepsia D07	0.78
NXYC157	*Lonicera japonica* Thunb	Han xue ni ba ze	Caprifoliaceae	Stem	W, C	L	Menstruation Irregular/Frequent X07, Rheumatoid Arthritis L88	0.39
NXYC230	*Morina nepalensis* D. Don var. delavayi (Franch.) C. H. Hsing	Yu he	Caprifoliaceae	Flower, Root	W	L	Cough R05, Anemia B82, Dyspepsia D07, Vaginal Discharge X14	0.61
NXYC261	*Nardostachys jatamansi* (D. Don) DC	Hua lei piao	Caprifoliaceae	Root	C	E	Epilepsy N88, Abdominal Pain D01, Abdominal Pain Epigastric D02, Teeth/Gum Disease D82, Dyspepsia D07	0.72
NXYC233	*Triplostegia grandiflora* Gagnep	Ju leng bu	Caprifoliaceae	Root	W	L	Anemia B82, Pain General/Multiple Sties A01, Poisoning by Medical Agent A84	0.61
NXYC012	*Valeriana jatamansi* Jones	Ruan ke ruan lei leng	Caprifoliaceae	Whole plant	W, C	L	Hepatitis D71, Chronic Enteritis/Ulcerative Colitis D94, Rheumatoid Arthritis L88	0.94
NXYC206	*Pittosporum heterophyllum* Franch	Che nu e	Pittosporaceae	Bark	W	L	Rheumatoid Arthritis L88, Menstruation Irregular/Frequent X07, Jaundice D13, Worms/other Parasites D96, Palpitation K04, Pain General/Multiple Sties A01, Peptic Ulcer D86, Post-partum Symptom/Complaint other W18, Bleeding/Haemorrhage Nos A10, Trauma A80	0.72
NXYC101	*Eleutherococcus nodiflorus* (Dunn) S. Y. Hu	Ban wu na e	Araliaceae	Bark	W	L	Rheumatoid Arthritis L88, Pain General/Multiple Sties A01, Abdominal Hernia other D91, Pruritus S02, Neurasthenia P78	0.83
NXYC269	*Eleutherococcus trifoliatus* (L.) S. Y	Ban wu na	Araliaceae	Stem, Bark	W	L	Rheumatoid Arthritis L88, Pain General/Multiple Sties A01, Parotitis R99	0.61
NXYC142	*Hedera nepalensis* var. sinensis (Tobl.) Rehd	Zuo teng	Araliaceae	Stem	W	L	Rheumatoid Arthritis L88, Menstruation Irregular/Frequent X07, Breast/Lactation Symptom W19, Hemorrhoids K96, Bronchitis R78, Pruritus S02	0.67
NXYC239	*Panax ginseng* C. A. Mey	Hong shen	Araliaceae	Rhizome	W, C	E	Menstruation Irregular/Frequent X07, Diabetes Mellitus T90, Dyspepsia D07, Palpitation K04	0.94
NXYC010	*Angelica sinensis* (Oliv.) Diels	Yun gui	Apiaceae	Whole plant	C	L	Menstruation Irregular/Frequent X07, Post-partum Symptom/Complaint other W18, Constipation D12, Pain General/Multiple Sties A01	1
NXYC009	*Bupleurum candollei* Wall	Mu ru	Apiaceae	Whole plant	W	L	Hepatitis D71, Dyspepsia D07, Influenza R80	0.44
NXYC109	*Heracleum candicans* Wall. ex DC	Chi nu ken	Apiaceae	Root	W	L	Rheumatoid Arthritis L88, Influenza R80	0.94
NXYC141	*Heracleum likiangense* Wolff	Ji jiao qi	Apiaceae	Root	W	L	Abdominal Pain Epigastric D02, Sinusitis R75	0.72
NXYC011	*Ligusticum chuanxiong* Hort	Xiong qiong	Apiaceae	Whole plant	C	L	Rheumatoid Arthritis L88, Menstruation Irregular/Frequent X07, Liver Disease NOS D97, Pain General/Multiple Sties A01	1
NXYC172	*Pimpinella candolleana* Wight et Arn	Yu ma zhe nu ken	Apiaceae	Root	W	L	Influenza R80, Rheumatoid Arthritis L88, Skin Disease Other S99, Abdominal Pain Epigastric D02, Tetanus N72	0.67
NXYC193	*Seseli yunnanense* Franch	Zhu ye fang feng	Apiaceae	Root	W	L	Influenza R80, Abdominal Pain D01, Neoplasm A21, Trauma A80	0.78

^a^ W/C: W, wild; C, cultivated

^b^E/L: E, Exotic; L, local

Angiosperms are sorted according to APG IV@@@

Identifying medicinal material with high UF values indicated its abundant use and widespread knowledge among the local communities [28]. In the present study, the UF ranged between 0.3 and 1 (Table 1, Table 2). Among the samples, there were 25 species (8.99%) with a UF value of 1, such as *Achyranthes bidentata*, *Aconitum carmichaelii*, *Rodgersia sambucifolia*, etc., 123 species (44.24%) with a UF value above 0.8, and 267 species (96.04%) with a UF value above 0.5, which showed that most of the medicinal materials sold in the market had a high degree of identification and utilization and were also commonly used medicinal materials.

**Table 2:** Use frequency (UF) values of 277 plants in this study

UF	Number	Proportion (%)
0.9–1.0	65	23.38
0.8–0.9	58	20.86
0.8–0.7	67	24.10
0.7–0.6	57	20.50
0.5–0.6	20	7.19
0.4–0.5	7	2.52
0.3–0.4	4	1.44

### Medicinal parts

**Fig. 4:**
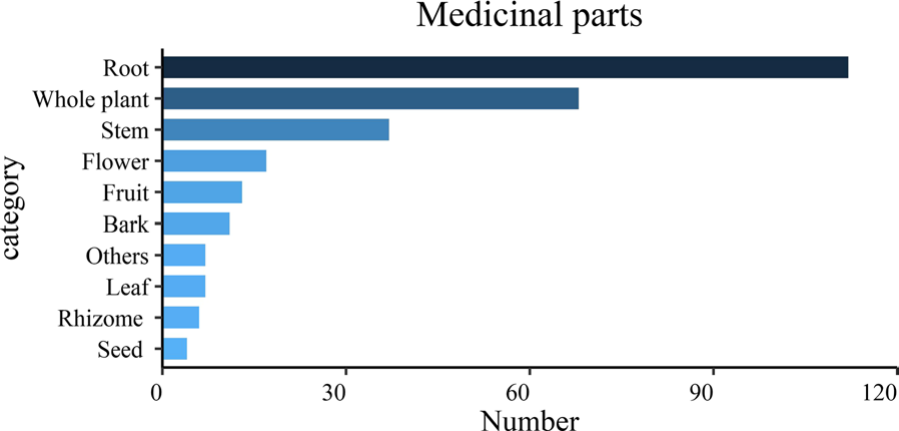
Numbers of medicinal plants belonging to different medicinal parts

In this market survey, the types of medicinal parts included the root and rhizome, stem, bark, leaf, fruit, seed, whole plant, flower and others, all reflecting the diversity of medicinal parts of plants used by the Naxi people in Lijiang. The root and rhizome are the main organs for organic storage. There were 112 species of medicinal plants from the root, accounting for 40.43% of the total number of species investigated, followed by the whole plant, with 68 species, accounting for 24.55%; by contrast, there were relatively few leaves and seeds (Fig. 4). These data were similar to those of previous studies [29]. However, the root and whole plants from wild plants are not conducive to the sustainable development of medicinal plants, so it is necessary to promote artificial planting.

### Medicinal applications and ICF

According to the ICPC-2, the herbs sold at the herbal market were used to treat 83 human ailments, which were divided into 16 categories (Table 3). This type of medicine can have multiple therapeutic uses. For example, *Oxalis corniculata* can be used to treat musculoskeletal system and connective tissue diseases, gynaecological system diseases and skin and subcutaneous tissue diseases. This variety shows the diversity of herbs for use by the Naxi people in the Lijiang area. Among the 16 medical categories, most medicinal materials were used to treat diseases of the digestive system (59.93%), followed by diseases of the general and unspecified system (57.04%), respiratory (46.21%), female genital (42.24%), skin (39.35%) and musculoskeletal systems (34.30%). Among the medicinal plants provided by different respondents, there are very few (only one or none) identical plants that can be used to treat the same group of diseases. This observation showed that there are many differences among the Naxi people in the methods for treating a specific disease, i.e., that they have low consensus about disease treatment methods. There are two possible reasons for this: (1) because the Naxi people live in biodiversity-rich areas, the abundant medicinal plant resources provided them with a wide choice of medicinal plants to use [30], and (2) different Naxi folk healers may have different degrees of understanding of the same disease.

**Table 3:** Informant consensus factor (ICF) values of the medicinal plants

Disease types	Number	Disease	The sum of plant species (Nur)	The number of identical plant species used (Nt)	ICF
Digestive	166	Worms/other Parasites D96	10	2	0.89
		Abdominal Hernia other D91	6	1	1.00
		Jaundice D13	22	2	0.95
		Hepatitis D71	40		1.03
		Vomiting D10	4	1	1.00
		Diarrhea D11	33	16	0.53
		Dyspepsia D07	30	7	0.79
		Chronic Enteritis/Ulcerative Colitis D94	17		1.06
		Constipation D12	9	0	1.13
		Liver Disease NOS D97	11	0	1.10
		Teeth/Gum Disease D82	13	2	0.92
		Haematemesis D14	8	3	0.71
		Hematochezia D16	5	3	0.50
		Peptic Ulcer D86	8	0	1.14
		Malignant Neoplasms Stomach D74	2	0	2.00
		Abdominal Pain Epigastric D02	29		1.04
		Appendicitis D88	5		1.25
		Abdominal Pain D01	6	0	1.20
		Perianal Abscess D95	1	0	-
General and unspcified	158	Pain General/Multiple Sties A01	99	27	0.73
		Bleeding/Haemorrhage Nos A10	25	4	0.88
		Malaria A73	10	3	0.78
		Tuberculosis A70	33	1	1.00
		Infectious Disease A78	3	0	1.50
		Trauma A80	25	5	0.83
		Poisoning by Medical Agent A84	7	2	0.83
		Neoplasm A21	22	4	0.86
Respiratory	128	Bronchitis R78	24	4	0.87
		Cough R05	28	5	0.85
		Influenza R80	55	14	0.76
		Tonsillitis R76	14	2	0.92
		Pneumonia R81	6	3	0.60
		Whooping Cough R71	6	0	1.20
		Foreign Body Nose/Larynx/Bronchus R87	1	0	-
		Upper Respiratory Infection R74	13	3	0.83
		Tracheitis R77	4	0	1.33
		Sinusitis R75	4	0	1.33
		Parotitis R99	13	2	0.92
Female genital	117	Menstruation Irregular/Frequent X07	74	8	0.90
		Breast Lump/Mass Female X19	8	1	1.00
		Breast Symptom Female other X21	17	2	0.94
		Vaginal Discharge X14	27	3	0.92
		Benign Neoplasm Female Genital X80	1	0	-
Skin	109	Boil Carbuncle S10	10	2	0.89
		Rash Localized S07	8	0	1.14
		Burns/Scald S14	12	1	1.00
		Pruritus S02	6	2	0.80
		Skin Disease Other S99	78	11	0.87
Musculoskeletal	95	Rheumatoid Arthritis L88	96	10	0.91
Urological	57	Dysuria U01	12	1	1.00
		Urinary Infection U71	17	3	0.88
		Urinary Calculi U95	7	1	1.00
		Pyelonephritis U70	34	3	0.94
		Cystitis U71	9	0	1.13
Cardiovascular	45	Hemorrhoids K96	14	3	0.85
		Heart Pain K01	8	0	1.14
		Hypertension K25	10	1	1.00
		Palpitation K04	6	1	1.00
		Stroke K90	9	0	1.13
Pregnancy, childbearing, family planning	31	Post-partum Symptom/Complaint other W18	16	0	1.07
		Breast/Lactation Symptom W19	6	0	1.20
		Abortion Spontaneous W82	7	1	1.00
		Infertility Female W15	6	0	1.20
		Question of Pregnancy W01	4	0	1.33
Neurological	25	Epilepsy N88	12	0	1.09
		Migraine N89	1	0	-
		Tetanus N72	4	2	0.67
		Meningitis N71	2	0	2.00
		Dizziness N17	4	1	1.00
Male genital	21	Prostatitis Y73	4	0	1.33
		Pain in Testis/Scrotum Y02	1	0	-
		Impotence Y07	16	0	1.07
		Syphilis Y70	4	0	1.33
Psychological	15	Dementia P70	2	0	2.00
		Sleep Disturbance P06	7	1	1.00
		Neurasthenia P78	6	0	1.20
Eye	14	Cataract F92	8	1	1.00
		Conjunctivitis F70	9	3	0.75
Endocrine/metabolic and nutritional	13	Diabetes Mellitus T90	12	1	1.00
		Lipid Disorder T93	1	0	-
Ear	7	Deafness H86	6	0	1.20
		Otitis Media H74	1	1	-
Blood, blood forming organs and immune mechanism	6	Anemia B82	7	3	0.67

The informant consensus factor (ICF) is a measure of information diversity. The higher the ICF value is, the greater the difference among plant species used in the treatment of a given disease, and the lower the ICF value is, the smaller the difference among plant species used in the treatment of a disease [25]. The highest ICF values were recorded in this study for meningitis N71, malignant neoplasms stomach D74, dementia P70 (ICF = 2.0) and infectious disease A78 (ICF = 1.50), followed by tracheitis R77, sinusitis R75, question of pregnancy W01, prostatitis Y73 and syphilis Y70 (ICF = 1.33). Further analysis indicated that most of the plant species were used for pain general/multiple sites A01 (Nur = 99, Nt = 27), followed by rheumatoid arthritis L88 (Nur = 96, Nt = 10), skin disease other S99 (Nur = 78, Nt = 11) and menstruation irregular/frequent X07 (Nur = 74, Nt = 8). These values indicated that these four groups of diseases are common in areas were the Naxi people live, and Naxi folk healers have a high consensus on the treatment of these diseases.

### Herbal medicine recorded in the Dongba Sutra

**Fig. 5:**
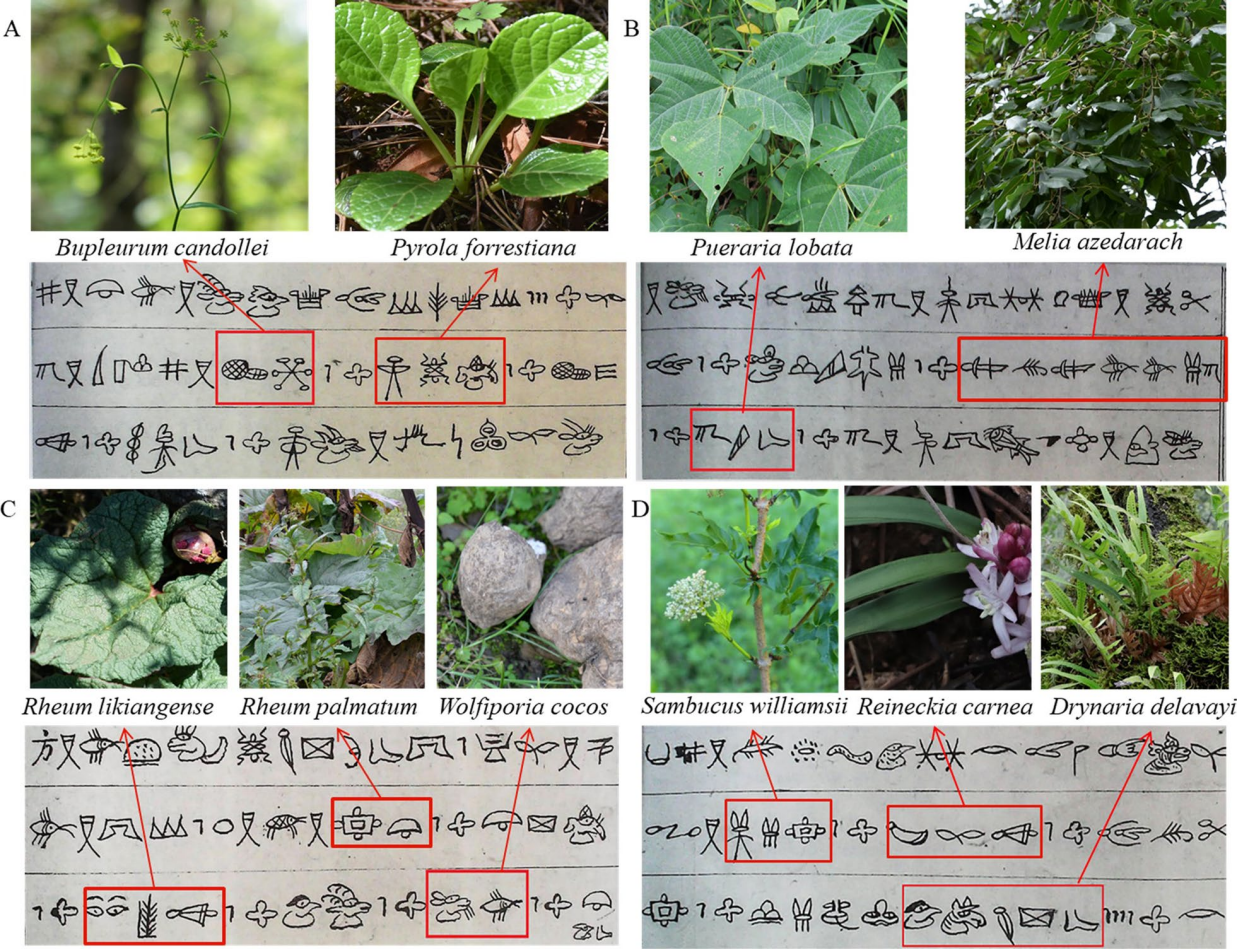
Some medicinal plants sold in the market and recorded in the Dongba Sutra

The Dongba Sutra recorded topics such as philosophy, history, religion and medicine and is used as a type of encyclopaedia for the Naxi community. Among the topics, many medical classics reflect the contents related to life and health in ancient times, which are the simple understanding of life, health and medicine of ancient people and were of great significance to the study of the origin of medicine. The Dongba people who practiced the primitive religion of the Naxi people mastered Dongba words and accumulated the initial knowledge and long-term practice of medicine [15]. In addition, they formed unique diagnosis and treatment theories and developed valuable experience in disease prevention and treatment. In this market research, 19 of 277 medicinal materials were recorded in the Dongba Sutra (Table 4, Fig. 5). All the medicines recorded in the Dongba Sutra are formulas, and the Naxi Dongba is compatible with medicines used to treat diseases. For example, to treat serious colds, the Naxi Dongba uses *Bupleurum candollei* and *Pyrola forrestiana* (Fig. 5A). *Pueraria lobata* and *Melia azedarach* can be used to treat malnutrition in children (Fig. 5B), *Rheum palmatum*, *Rheum likiangense* and *Wolfiporia cocos* can be used to treat urinary infection (Fig. 5C), and *Reineckia carnea*, *Sambucus williamsii* and *Drynaria delavayi* can be combined to treat dyspepsia (Fig. 5D).

**Table 4:** Medicinal plants sold in the market and recorded in the Dongba Sutra

Voucher specimens	Local name	Latin name of original plant	International phonetic alphabet	Hieroglyphs
NXYC002	Chong beng	*Acorus calamus* L.	ʦhu^33^bɯ^21^khɯ^33^	
NXYC009	Mu ru	*Bupleurum candollei* Wall	ʦæ^21^hu^21^	
NXYC078	Ba qi	*Cirsium japonicum* Fisch. ex DC	ʐuɑ^33^ʨhi^21^khɯ^33^	
NXYC093	Lu ba di li	*Drynaria delavayi* Christ	huɑ^55^ʐuɑ^33^ʥi^21^ʦhe^33^khɯ^33^	
NXYC119	Me mu	*Engleromyces goetzii* P.Henn	mɯ^55^mu^55^	
NXYC029	Qie san che e	*Geranium strictipes* R.Knuth	kə^12^se^33^ɕiɑ^33^	
NXYC076	A you jian da ken	*Hemiphragma heterophyllum* Wall	ʂʅ^12^ʨi^33^ʦhɑ^33^	
NXYC003	Da lv lv	*Melia azedarach* L	dɑ^33^bɯ^33^dɑ^33^ly^55^ly^33^	
NXYC077	Leng gan se ji	*Oxalis corniculata* L	huɑ^33^ʨhi^21^	
NXYC020	Gan gan er	*Pueraria lobata* (Willd.)Ohwi	gɑ^55^do^21^khɯ^33^	
NXYC154	Guang huang cao	*Pyrola forrestiana* H.Andres	lu^12^hæ^21^ʦhɑ^33^	
NXYC117	Jiu jie ling	*Reineckea carnea* (Andrews) Kunth	gv^33^khɯ^21^gv^33^tʂər^55^zɿ^33^	
NXYC060	Xi pu gao nu na	*Rheum likiangense* Sam	ȵiə^21^ŋv^33^ʨhi^21^	
NXYC022	Hua zeng de	*Rheum palmatum* L	dɑ^55^huæ^21^	
NXYC044	Hua leng hua zeng ke	*Rumex nepalensis* Spreng	huɑ^55^ʦe^33^	
NXYC050	Wo zhu ze	*Sambucus williamsii* Hance	ʦie^12^gu^12^dæ^33^	
NXYC049	Guo ji lv	*Schisandra rubriflora* (Franch.) Rehd.et Wils	ko^21^lɯ^55^se^33^	
NXYC034	Ci lv lv ru da biao	*Selaginella pulvinata* (Hook. et Grev.) Maxim	ʦhɯ^33^ly^21^ly^21^ʐu^21^dɑ^333^biə^33^	
NXYC048	Tuo ken lv	*Poria cocos* (Schw.)Wolf	tho^33^khɯ^33^ly^33^

### Herbal medicine recorded in *Yulong Ben Cao*

**Fig. 6:**
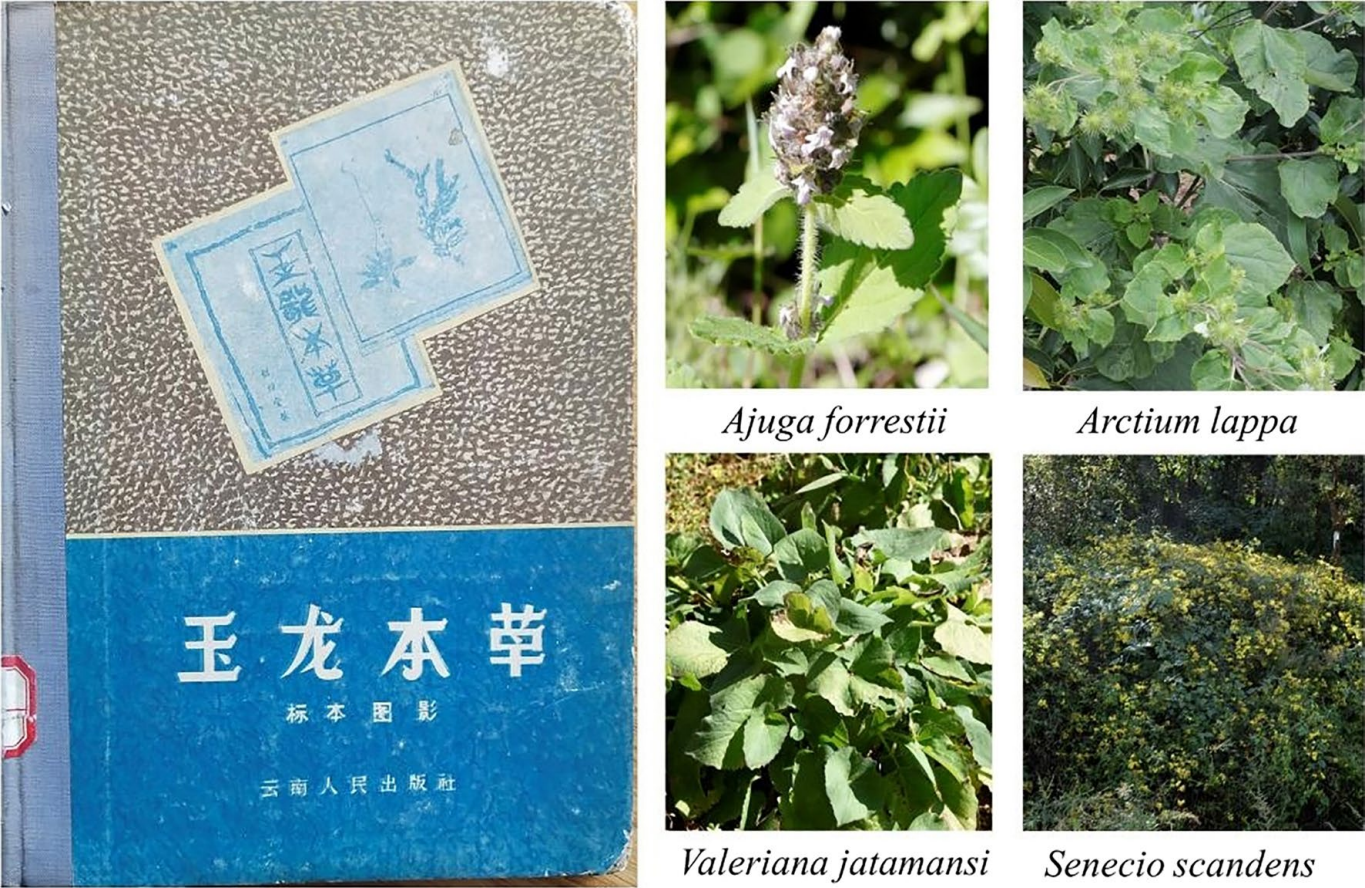
Some medicinal plants sold in the market and recorded in *Yulong Ben Cao*

**Fig. 7:**
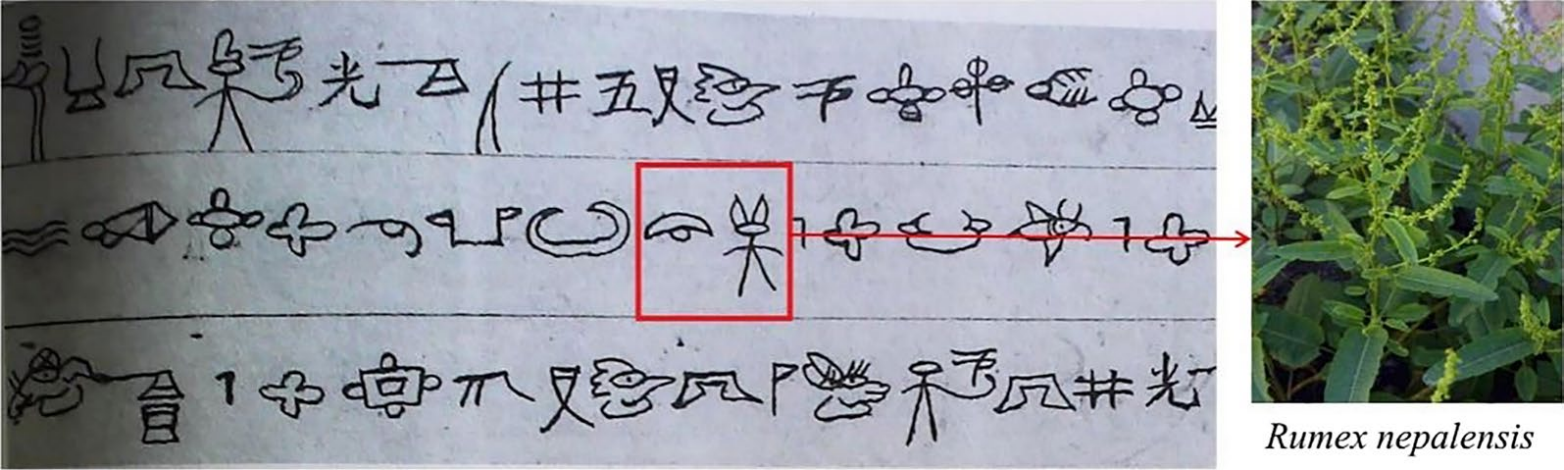
*Rumex nepalensis* as recorded in the Dongba sutra

The *Yulong Ben Cao* was compiled based on the environment, climate, eating habits and other diseases where the Naxi community is situated. It is a key source on Naxi medicine [31]. In this market research, 12 of the medicinal plants were recorded in the *Yulong Ben Cao* (Fig. 6), namely *Cynanchum otophyllum*, *Rodgersia sambucifolia*, *Swertia punicea*, *Geum aleppicum*, *Salvia trijuga*, *Polygala arillata*, *Senecio scandens*, *Polygonum paleaceum*, *Rumex nepalensis*, *Arctium lappa*, *Ajuga forrestii* and *Valeriana jatamansi*. Among these species, *Rumex nepalensis* is also recorded in the Dongba Sutra. In the Dongba Sutra, *Rumex nepalensis* is mashed, mixed with honey and smashed green onions to treat men with hernia (Fig. 7). In the *Yulong Ben Cao*, the indications for *Rumex nepalensis* are different, and it is primarily used for treating skin eczema, sweat spots, acute tonsillitis, constipation and other ailments [32]. In the theory of TCM [33], the indications for *Rumex nepalensis* are roughly the same as those recorded in *Yulong Ben Cao*. The *Yulong Ben Cao* was written in Chinese by an author from the Naxi people and is the product of the combined culture of Naxi medical culture and Han medical culture [34]. Lijiang is in north-western Yunnan Province at the junction of Yunnan, Sichuan and Tibet. It is a multi-ethnic place. In addition to Han and Naxi, there are 21 ethnic minorities, such as Tibetan, Bai and Yi. Some medicinal materials are also used by these ethnic minorities, such as *Rumex nepalensis*. Tibetans use it to treat sores and ulcers, as recorded in the Jingzhu Materia Medica [35]. The Bai people use it to treat constipation, gastrointestinal haemorrhage, eczema, etc. [36]. The Naxi people also have this usage [32]. These phenomena reveal the interrelationship between the Naxi medical culture and the medical culture of the surrounding ethnic groups.

### Resources status

**Fig. 8:**
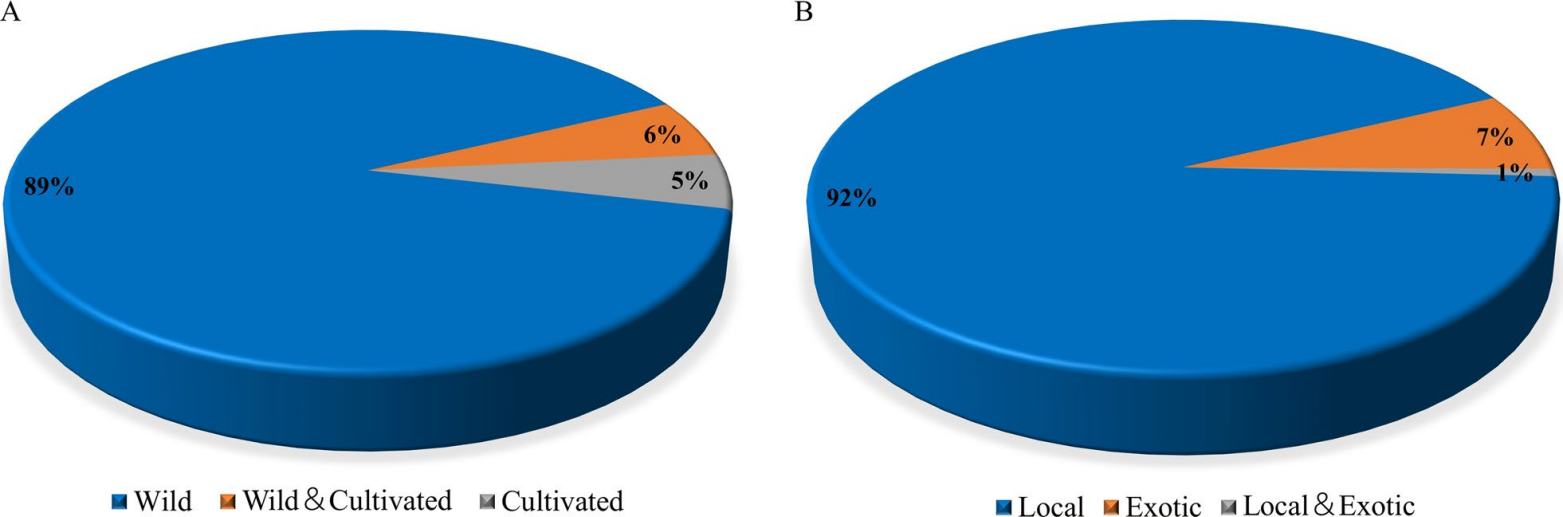
**A** Wild cultivation of medicinal plants on the markets. **B** Sources of medicinal plants on the markets

Among the original plants from the 277 species of medicinal materials, 266 (92.39%) were from the local area, and 21 (6.92%) were from other places. Among them, 258 species (89.27%) were completely wild, 15 species (5.19%) were cultivated, and 16 species (5.54%) were wild or cultivated (Fig. 8). This observation was consistent with the trend in the survey results from the Honghe area of Yunnan Province, where wild medicinal plants accounted for 80.1% [9]. The possible reason was that most of the sellers surveyed during the two surveys were local rhizotomists.

According to the ISCREP [21], 2 of the 277 medicinal materials were listed as first class nationally protected plants in China (Table 5), and they were *Tiepishihu* and *Renshen*. This finding was also consistent with a survey of the medicinal materials market in Dali Prefecture, Yunnan [13]. According to the IUCN Red List of Threatened Species, *Ginkgo biloba* and *Panax ginseng* are "critically endangered" species. *Dendrobium officinale* was not recorded. Fortunately, both *Ginkgo biloba* and *Panax ginseng* can be obtained through cultivation. There were 17 species listed as second-class nationally protected plants in China (Table 5), of which 15 species sold in the markets of Lijiang were completely taken from wild plants. It is worth noting that *Psammosilene tunicoides*, *Bletilla striata*, *Gymnadenia conopsea* and *Rhodiola crenulata* were classified as "endangered" species according to the IUCN Red List of Threatened Species, but all those currently sold on the Lijiang market were collected from the wild; *Dysosma versipellis* and *Gymnadenia orchidis* belong to the "vulnerable" species according to the IUCN Red List of Threatened Species, and all those sold in the Lijiang markets were also collected from the wild. Although *Paris pubescens* and *Cistanche deserticola* were classified as "endangered" species according to the IUCN Red List of Threatened Species, they could be cultivated and obtained at present. Reasonable utilization and effective protection for these wild species are extremely vital; otherwise, they might be endangered in the near future.

**Table 5:** First class nationally protected plants and second class nationally protected plants

Voucher specimens	Local name	Family name	Latin name of original plant	W/C	L/E	IUCN
NXYC175	Ai shi ban mi ba	Orchidaceae	*Dendrobium officinale* Kimura et Migo	W	L	–
NXYC239	Hongshen	Araliaceae	*Panax ginseng* C. A. Mey	W/C	E	CR
NXYC024	Me ji xu	Crassulaceae	*Rhodiola fastigiata* (Hook. f. et Thoms.) S. H. Fu	W	L	LC
NXYC043	Lv ji piao	Orchidaceae	*Bulbophyllum odoratissimum* (Smith) Lindl	W	L	LC
NXYC054	Ke ba guo lian	Berberidaceae	*Dysosma versipellis* (Hance) M. Cheng ex Ying	W	L	VU
NXYC085	Fen cao	Fabaceae	*Glycyrrhiza uralensis* Fisch	C	E	LC
NXYC140	A dong ming	Orchidaceae	*Calanthe tricarinata* Lindl.ex Wall	W	L	LC
NXYC150	Da du wu	Crassulaceae	*Rhodiola yunnanensis* Franch	W	L	LC
NXYC162	Xiao ban ye lan	Orchidaceae	*Goodyera repens* (L.) R. Br	W	L	LC
NXYC177	Lei ke	Orchidaceae	*Gastrodia elata* Blume	W/C	L	–
NXYC213	Di yu	Caryophyllaceae	*Psammosilene tunicoides* W. C. Wu et C. Y. Wu	W	L	EN
NXYC219	Yu ma pu	Melanthiaceae	*Paris pubescens* (Hand.-Mazz.) Wang et Tang	W/C	L	EN
NXYC224	Gong ben ya de	Orchidaceae	*Bletilla striata* (Thunb.) Rchb. f	W	L	EN
NXYC225	Gong ben ya ji	Orchidaceae	*Pleione bulbocodioides* (Franch.) Rolfe	W	L	LC
NXYC236	A yu la ba	Orchidaceae	*Gymnadenia conopsea* (L.) R. Br	W	L	EN
NXYC237	Xinanshoushen	Orchidaceae	*Gymnadenia orchidis* Lindl	W	L	VU
NXYC240	Roucongrong	Orobanchaceae	*Cistanche deserticola* Y. C. Ma	C	E	EN
NXYC245	Wu lu me ji xu	Crassulaceae	*Rhodiola crenulata* (Hook. f. et Thoms.) H. Ohba	W	L	EN
NXYC247	Lu bu gei	Orchidaceae	*Spiranthes sinensis* (Pers.) Ames	W	L	LC

*CR* critically endangered, *LC* least concern, *EN* endangered, *VU* vulnerable

## Discussion

### The ecological ethics of Naxi people have positive significance for the conservation of wild plant resources

The study area, the Lijiang area in Yunnan Province of China, is well known for its exceptional richness in medicinal plants. We recorded 277 species of medicinal plants being traded on the markets involving 97 families, such as Asteraceae, Rosaceae and Ranunculaceae. The medicinal parts, including the roots and rhizomes, stems, skins, leaves, fruits, seeds, whole plants, flowers, etc., showed abundant plant diversity and rich local knowledge in this area. Yunnan Province is called “the kingdom of animals and plants”, for possessing extremely rich biological resources. There are many medicinal plants, especially the species in the Asteraceae, Ranunculaceae and Liliaceae. Systematic research on these key families would help to develop new medicinal resources and protect endangered species.

The ancestors of the Naxi people attached great importance to the harmonious development of man and nature. The Naxi people consider human beings and nature to be brothers. This ecological ethics concept laid the foundation for the Naxi people to live in harmony with nature; it shows the most primitive and simple concept of environmental conservation by human beings [37]. Experienced medicinal gatherers attach great importance to the sustainable use of resources. They generally pick large herbs rather than small herbs. Most of them collect herbs when the seeds are mature and sow seeds in the surrounding area to be able to renew them naturally [27]. However, with the increase in usage, the demand for trade in medicinal plants may increase in the coming years, leading to the over harvesting of wild plant species and possibly even endangering natural populations. In this study, we found that most of the medicinal plants use roots and whole plants, including the second-level nationally protected plants *Rhodiola fastigiata*, *Bulbophyllum odoratissimum*, *Dysosma versipellis*, *Spiranthes sinensis*, etc., and they are all wild plants. Over harvesting is not conducive to the sustainable development of these plants. The sustainable management of wild medicinal plants is very important to protecting their diversity and preventing their extinction, especially for species that are frequently used in traditional medicine. Therefore, on the one hand, we should strengthen the punishments for indiscriminate digging, and on the other hand, we should engage in the artificial domestication and cultivation of large and precious medicinal materials to alleviate the rapid decline in the current medicinal plant resources.

### Naxi medicinal plants are versatile and have local characteristics

In terms of disease treatment, the herbal applications by the Naxi people almost covers common diseases, including dyspepsia, the common cold, menstruation disturbances, fractures, etc. as well as incurable infectious diseases such as rabies and malaria in addition to current research hotspots such as cancer and cardiovascular diseases. Common local diseases, such as rheumatoid arthritis and external injury are also addressed. During the use of medicinal plants, a versatile feature is very common; for example, *Aconitum brachypodum* can be used to treat cancer and rheumatoid arthritis. *Toddalia asiatica* can be used to treat rheumatoid arthritis, external injury, gastrointestinal bleeding and menstruation disturbances.

The Naxi people constantly learned and absorbed the culture and production technology of the various surrounding ethnic groups while communicating with them, thereby enriching and developing their own culture and promoting their own national progress and development [23]. In the Dongba Sutra "Genesis" (Chuang Shiji), it is mentioned that the Tibetans Bai and Naxi were closely related. In the Naxi creation epic "Chongmo Chongze", it is also mentioned that the Naxi and the Tibetans are brothers [33]. This recording also showed that the Naxi people had close exchanges with Tibetans, Bai nationalities and other ethnic groups. In this study, we found that the Naxi, Bai and Tibetans also share similarities in medicinal plant applications. For example, both the Naxi and Tibetans use *Rheum palmatum* to treat constipation, dyspepsia, diarrhoea, jaundice, carbuncles, amenorrhea, etc. In addition, the Naxi people also use *Rheum palmatum* to treat vomiting, gastrointestinal haemorrhages, tumours and other diseases, and the Tibetans also use it to treat infectious diseases, fever and other diseases.

### Naxi medical classics record the excellent culture of the Naxi people

The "Dongba religion" is the most primitive religion of the Naxi people. Various cultural activities and phenomena, such as Dongba words, sutras, rituals and music making up the Dongba religion, are called the Dongba culture [38]. Many medicinal plants and their uses were recorded in the Dongba Sutra. In this market survey, a total of 19 medicinal plants were recorded in the Dongba Sutra, including *Bupleurum candollei*, *Pyrola forrestiana*, *Rheum palmatum*, etc. In the survey, 19 medicinal plants were all found to be from the local area of Lijiang. There were common medicinal materials used by people from ancient times to the present. The Naxi people have a long history of recognizing and using medicines.

The *Yulong Ben Cao* was a local herbal book written under the guidance of traditional Chinese medicine theory and combined with the personal experience of the Naxi people [23]. In this market survey, a total of 12 herbs were recorded in the *Yulong Ben Cao*, including *Cynanchum otophyllum*, *Rodgersia sambucifolia* and *Swertia punicea*. Some of them were also recorded in the Dongba Sutra, such as *Rumex nepalensis*. However, the usage of *Rumex nepalensis* in the *Yulong Ben Cao* is different from that in Dongba Sutra. The usage of *Rumex nepalensis* in the *Yulong Ben Cao* is closer to that of TCM. Thus, Naxi medicine has absorbed the practice and theory of TCM to promote the formation and development of a national medicine.

The development of Naxi medicine has gone through a long historical process. There are records of medical knowledge in many ancient Dongba Sutra works. With the integration of various ethnic cultures, Naxi medicine has been deeply influenced by various cultures, such as Han, Tibetan and Bai. Unlike other ethnic medicines, Naxi medicine is a multicultural medical theory that has absorbed the practices and theories of TCM, Tibetan medicine and other ethnic medicines and combines the characteristics of its own ethnicity.

## Conclusion

This research is the first contribution toward understanding, from an ethnobotanical point of view, that medicinal plants play an important role in the lives of the Naxi people. We studied the records of medicinal plants sold in the markets in the Dongba Sutra and *Yulong Ben Cao*. The traditional knowledge of medicinal plants recorded in these medical classics is the result of ancient humans' understanding of nature. From the perspective of the relationship between humans and nature, the content they contain has important ethnobotanical value. However, traditional medicine knowledge and medicinal plants are greatly threatened by rapid economic development for various reasons. Therefore, policies and practices to protect medicinal plants and their associated traditional knowledge are necessary.

## Data Availability

All the data generated or analysed during this study are included in this published article (and its supplementary information files).

## Abbreviations

ICF: Informant consensus factor
UF: Use frequency
ICPC-2: International Classification of Primary Care
ISCREP: Information System of Chinese Rare and Endangered Plants
TCM: Traditional Chinese Medicine
CR: Critically endangered
LC: Least concern
EN: Endangered
VU: Vulnerable
